# Multi-omics dissection of MAPK-driven senescence unveils therapeutic vulnerabilities in KIAA1549::BRAF-fusion pediatric low-grade glioma models

**DOI:** 10.1038/s41392-025-02279-8

**Published:** 2025-06-23

**Authors:** Romain Sigaud, Anja Stefanski, Florian Selt, Daniela Kocher, Diren Usta, Daniel Picard, Isabel Büdenbender, Marc Remke, Stefan M. Pfister, David T. W. Jones, Tilman Brummer, Olaf Witt, Till Milde

**Affiliations:** 1 Department of Pediatrics and Adolescent Medicine, Jena University Hospital, Friedrich Schiller University Jena, Jena, Germany; 2 Comprehensive Cancer Center Central Germany (CCCG), Jena, Germany; 3 Hopp Children’s Cancer Center Heidelberg (KiTZ), Heidelberg, Germany; 4 Clinical Cooperation Unit Pediatric Oncology, German Cancer Research Center Heidelberg (DKFZ), Heidelberg, Germany; 5 Molecular Proteomics Laboratory (MPL), Biological-Medical Research Center (BMFZ), Heinrich-Heine University Düsseldorf, Düsseldorf, Germany; 6 National Center for Tumor Diseases (NCT), Heidelberg, Germany; 7 Department of Pediatric Oncology, Hematology, Immunology and Pulmonology, Heidelberg University Hospital, Heidelberg, Germany; 8 Faculty of Biosciences, Heidelberg University, Heidelberg, Germany; 9 Center for Cancer Cell Therapy, Stanford Cancer Institute, Stanford University School of Medicine, Stanford, CA USA; 10 Department of Pediatric Hematology and Oncology, University Medical Center of Saarland, Homburg/Saar, Germany; 11 Department of Pediatric Neuro-Oncogenomics, German Cancer Research Center (DKFZ), Heidelberg, Germany; 12 German Consortium for Translational Cancer Research (DKTK), partner site Essen/Düsseldorf, Düsseldorf, Germany; 13 Department of Pediatric Oncology, Hematology, and Clinical Immunology, Medical Faculty, University Hospital Düsseldorf, Düsseldorf, Germany; 14 Department of Neuropathology, Medical Faculty, Heinrich-Heine University Düsseldorf, Düsseldorf, Germany; 15 Division of Pediatric Neurooncology, German Cancer Research Center (DKFZ) and German Consortium for Translational Cancer Research (DKTK), Heidelberg, Germany; 16 Division of Pediatric Glioma Research, German Cancer Research Center (DKFZ), Heidelberg, Germany; 17 Institute of Molecular Medicine and Cell Research (IMMZ), Faculty of Medicine, University of Freiburg, Freiburg, Germany; 18 Centre for Biological Signaling Studies BIOSS, University of Freiburg, Freiburg, Germany; 19 German Consortium for Translational Cancer Research (DKTK), Freiburg, Germany and German Cancer Research Center (DKFZ), Heidelberg, Germany

**Keywords:** Paediatric cancer, CNS cancer, Senescence, Drug screening

## Abstract

Pilocytic astrocytomas (PA), the most common pediatric low-grade gliomas (pLGGs), are characterized by genetic MAPK pathway alterations leading to constitutive activation and oncogene-induced senescence (OIS) accompanied with the senescence-associated secretory phenotype (SASP). This study investigates the molecular mechanisms of signaling pathways regulating OIS and SASP in pLGGs using a multi-omics approach. We utilized senescent DKFZ-BT66 cells derived from a primary KIAA1549::BRAF-fusion positive PA to generate RNA-sequencing and phospho-/proteomic datasets before and after treatment with the MEK inhibitor trametinib. Multi-omics factor analysis (MEFISTO) and single sample gene set enrichment analysis (ssGSEA) were employed to identify key OIS effectors and differentially regulated pathways upon MAPK inhibition. Trametinib treatment inhibited MAPK activity, OIS and SASP signatures across all omics levels, functionally underscored by reduced sensitivity towards senolytic drugs. We constructed a pathway network using a prior knowledge approach, mapping *n* = 106 upregulated and *n* = 84 downregulated direct downstream effectors of MAPK leading to OIS/SASP. These effectors are associated with better progression-free survival in pLGG patients, independent of tumor site, level of resection, and genetic aberration. Several compounds targeting signaling nodes (SOD-1, IRS1, CDK1/2, CK2) involved in OIS and under MAPK control were identified, of which *n* = 4 were validated in an additional primary KIAA1549::BRAF fusion pLGG model as potential new therapeutic vulnerabilities for the treatment of pLGG. Our unbiased multi-omics signaling pathway analysis identifies a specific and comprehensive network of MAPK-OIS-SASP interdependencies in pLGGs and suggests new therapeutic strategies for these tumors.

## Introduction

Pediatric low-grade gliomas (pLGGs) are the most common brain tumors in children, with pilocytic astrocytoma, its most common entity, accounting for approximately 20% of all primary brain and central nervous system tumors.^1^ These tumors are characterized by a low proliferation index.^2^ and a 10-year overall survival of around 90%, synonymous with a generally favorable prognosis.^3^ However, their location in critical areas of the brain can lead to significant morbidity,^4^ and some patients experience tumor progression or recurrence despite current treatments.^5,6^ The Mitogen-activated protein kinase (MAPK) pathways, particularly the RAF-MEK-ERK signaling cascade, play a crucial role in cell proliferation, differentiation, and survival.^7^ In pLGG, virtually all driving alterations occur in this signaling pathway,^8–10^ with the most common being the tandem duplication leading to a KIAA1549::BRAF fusion.^11^

A main feature of pLGG tumors is oncogene-induced senescence (OIS),^12–14^ a cellular state triggered by oncogenic activation that serves as a tumor-suppressive mechanism.^15^ OIS is often accompanied by the senescence-associated secretory phenotype (SASP),^16,17^ which involves the secretion of various factors that can sustain OIS in surrounding cells in a paracrine manner,^18^ and influence the tumor microenvironment.^19^

While OIS and SASP are thought to be regulated by the MAPK pathway, the complex interplay between MAPK signaling, OIS, and SASP in pLGGs remains elusive. While studies showed associations between BRAF overexpression and increased expression of a few senescence-associated molecules (p16, p21) in artificial models,^12,20^ little is known about the molecular downstream effects of MAPK activation in pLGG leading to OIS/SASP.

In this study, we aimed to elucidate the sequential molecular mechanisms controlling OIS and SASP in pLGGs and their regulation by the MAPK pathway. We employed a multi-omics approach using the patient-derived senescent pLGG cell model DKFZ-BT66, treated with the MEK inhibitor trametinib. The DKFZ-BT66 cells are transduced with a doxycycline-inducible vector controlling the expression of the SV40 large T antigen, which inhibits p53 and pRB.^13^ Upon doxycycline treatment, OIS is bypassed and cells grow in vitro. Removing the doxycycline halts SV40 large T antigen production, reinstating the original OIS phenotype. In the present study, we used the pLGG cells in their senescent state, with the objectives to (1) generate a pLGG-specific comprehensive multi-omics dataset to validate and study the effects of the MAPK pathway on OIS and SASP programs in pLGG cells, (2) identify novel interdependencies, putative targets and co-regulated pathways, both involved in OIS/SASP as well as regulated by the MAPK pathway, and (3) screen a panel of clinically relevant drugs targeting these newly identified molecules to validate these new vulnerabilities in pLGG cells.

By addressing these objectives, we aimed to provide a signaling network resource, new insights into the molecular underpinnings of pLGGs and identify potential therapeutic strategies for these tumors. Our findings contribute to developing more effective and targeted treatments for pediatric patients with low-grade gliomas.

## Results

### Generation of a multi-omics dataset using the PA-derived senescent DKFZ-BT66 cell line treated with trametinib

**Fig. 1:**
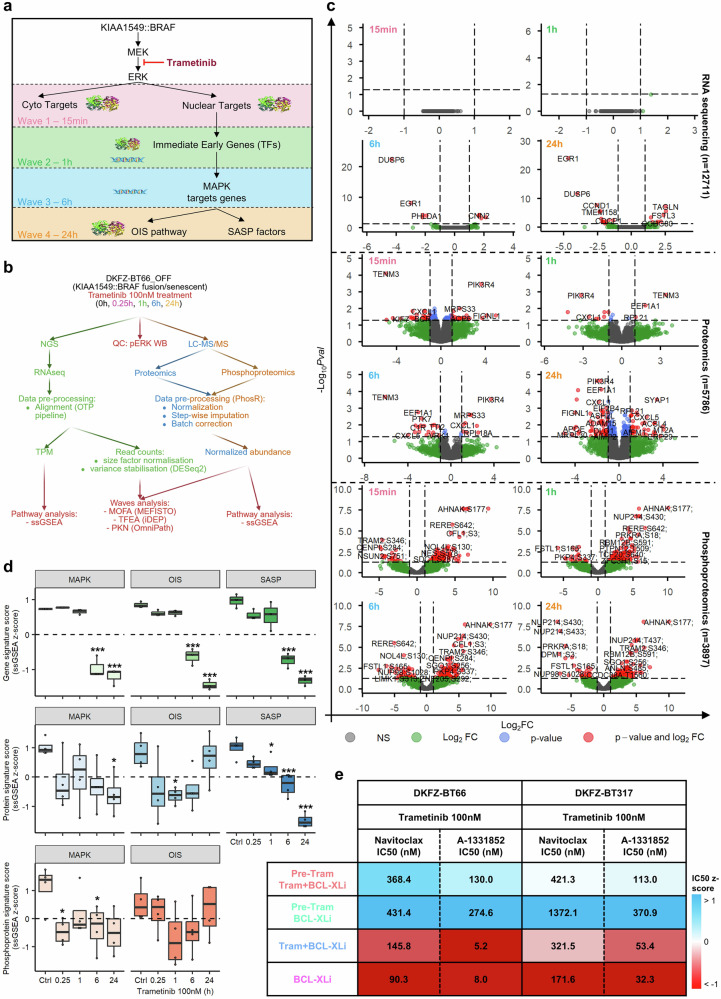
MAPKi reverses OIS molecular and cellular effect in pLGG cell lines. **a** Schematic depiction of the MAPK signaling pathway, and the strategy of sequential inhibition of the pathway with trametinib 100 nM for different time span to identify key molecules differentially regulated upon MAPKi for each wave of activation. **b** Graphical depiction of the bioinformatics pipeline used in the course of the study. **c** Volcano plots depicting the differentially regulated mRNA, proteins and phosphopeptides identified in the senescent DKFZ-BT66 cells after 100 nM trametinib treatment. **d** Boxplot depicting the ssGSEA z-score of MAPK-/OIS-/SASP-related signatures, specific for RNAseq, proteomics and phosphoproteomics, in senescent DKFZ-BT66 cells treated with 100 nM trametinib for the indicated time span. Boxplots depict the median, first and third quartiles. Whiskers extend from the hinge to the largest/smallest value no further than 1.5 * IQR from the hinge (where IQR is the inter-quartile range). Significance was calculated with one-way ANOVA followed by Dunnett’s multiple comparisons test in Prism. *** adj. p-val < 0.001, not significant if not specified. *n* = 3 independent biological replicates were used for the RNAseq samples, and *n* = 4 independent biological replicates for the phospho-/proteomics samples. **e** Summary heatmap of the corresponding senolytic IC_50_ in the senescent DKFZ-BT66 and senescent DKFZ-BT317 in the different treatment schedule. The results depict the average IC_50_ of *n* = 3 independent biological replicates. Shading depicts the concentrations z-score calculated across rows, while the values in the heatmap represent raw IC_50_ in nM

DKFZ-BT66 cells in the senescent state were treated with the MEK inhibitor (MEKi) trametinib (100 nM) for different time spans in order to sequentially shut down the MAPK pathway and identify the key molecules involved in the pathway’s waves of activation (Fig. 1a). Time points were selected based on the differential regulation of the direct downstream substrate (pERK – 15 min), immediate early gene (FOS – 1 h) and target gene (IL-1B – 6 h) expression, while 24 h was used as final time point where all waves were completely downregulated (supplementary Fig. 1a and supplementary Table 1). For each time point, RNAseq, proteomics and phosphoproteomics samples were generated and used to proceed with further bioinformatic analyses (Fig. 1b). Western blot analysis validated the MAPK inhibition in the generated samples (supplementary Fig. 1b). In total, *n* = 12,711 genes, *n* = 5 786 proteins and *n* = 3 897 phosphopeptides were found differentially regulated upon MAPKi across all time points (*p* < 0.05, |log2FC | > 1) (Fig. 1c and supplementary Table 2).

### MAPK pathway inhibition reduces oncogene-induced senescence and SASP programs on the gene, protein and phosphoprotein level, and desensitizes pLGG cells to senolytics treatment

The enrichment analysis of gene, protein, and phosphoprotein specific signatures (supplementary Table 3) showed a significant inhibition of the MAPK pathway and OIS upon trametinib treatment on all three omics levels, while a significant inhibition of the SASP was observed on the gene expression level, and a trend towards inhibition on the protein level. A trend for the OIS signature going back to normal after 24 h was observed on the protein and phosphoprotein level only. Of note, no SASP signature for the phosphoproteomics layer was used due to the lack of such a signature (Fig. 1d and supplementary Table 4). Altogether, this suggests a connection between MAPK activity and the OIS/SASP processes. Only minimal overlap was observed between the MAPK/OIS/SASP signatures for each omics layers, ruling out a potential confounding bias by overlapping genes (supplementary Fig. 1c). A co-treatment experiment combining sequential and/or simultaneous MAPK inhibition and senolytic treatment was carried out in the senescent DKFZ-BT66 and DKFZ-BT317 (both expressing a KIAA1549::BRAF fusion) to assess the effect of MAPKi inhibition on the sensitivity of pLGG cells to the senolytic drugs (BCL-XLi) (supplementary Fig. 1d). While a direct co-treatment of 100 nM trametinib and varying concentrations of navitoclax or A - 1331852 (both BCL-XLi) gave IC_50_ similar to each BCL-XLi alone, pre-treatment with 100 nM trametinib for 24 h increased IC50 of each BCL-XLi significantly in one model (DKFZ-BT317) and with a trend towards significance in the second model (DKFZ-BT66) (Fig. 1e, supplementary Fig. 1e and supplementary Table 5). This increase brought the IC_50_ values to levels comparable to those observed in BCL-XL inhibitor-resistant BT40 cells (navitoclax IC_50_ = 462.2 nM, A-1331852 IC_50_ = 112.3 nM in BT40 cells treated in the same condition).^21^ Taken together, the data suggest that the MAPK pathway directly positively regulates the OIS/SASP program in a feed-forward manner in the senescent pLGG cells.

### Mapping of the molecular partners regulated by the MAPK pathway and involved in OIS/SASP highlights novel putative targets for pLGG

**Fig. 2:**
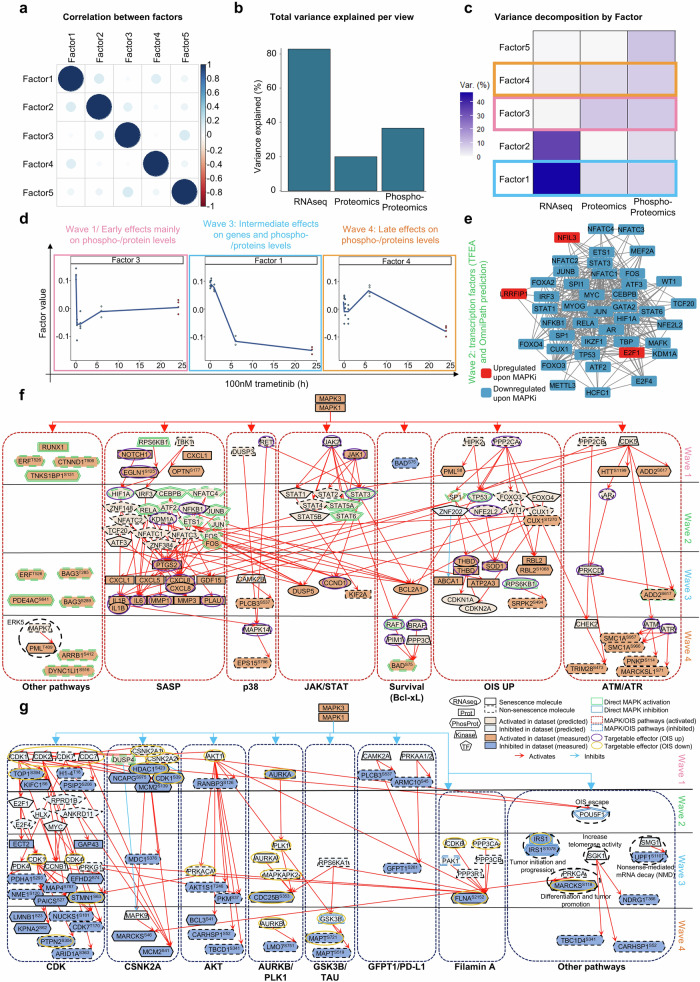
Mapping of the multi-omics MAPK/OIS/SASP axis in pLGG cells. **a** Dotplot depicting the degree of correlation between factors identified in the MEFISTO model. **b** Bar graph depicting the total variance explained per omics layer (=view). **c** Depiction of the variance decomposition per factor. The graph shows how much variance is explained by which omics layer, for each factor. **d** Dissection of the factors identified as related to the waves 1, 3, and 4. **e** Protein–protein interaction network of the transcription factors identified by TFEA as being involved in the regulation of the genes from wave 3 and 4. Blue nodes are downregulated upon MAPKi, red nodes are upregulated upon MAPKi. **f** Senescence signaling pathway involving all molecules upregulated in OIS and under the control of the MAPK pathway in the senescent DKFZ-BT66. **g** Senescence-related signaling pathways involving all molecules downregulated in OIS and under the control of the MAPK pathway in the senescent DKFZ-BT66. Node’s shape depicts what omics layer/analysis the effector was identified from

The pre-processed RNAseq, proteomics and phosphoproteomics datasets (supplementary Table 6) were first quality controlled via unsupervised hierarchical clustering to ensure that the filtering step prior integration still reflected the biological variations expected upon MAPKi through time (supplementary Fig. 2a). The data were then used to identify the downstream MAPK pathway effectors related to OIS/SASP using the method for the functional integration of spatial and temporal omics data (MEFISTO).^22^ The identified MAPK-regulated molecules were further filtered with the SeneQuest database^23^ to only retain molecules related to senescence. Finally, the connecting network was built using prior knowledge network interaction from the OmniPath database^24^ (supplementary Fig. 2b). MEFISTO identified *n* = 5 factors containing molecules differentially regulated upon MAPKi in similar fashion through time across all omics layers. All factors were largely uncorrelated (Fig. 2a), with the model explaining at least or more than 20% of the variation in each omics layers, suggesting a good fit of the model (Fig. 2b). By variance decomposition, Factor 1 explained most of the variance in the RNAseq level, while Factors 3 and 4 explained most of the variance in the proteomics and phosphoproteomics layers (Fig. 2c). Factor 3 was mostly regulated after 15 min reflecting the early events on the phospho-/protein levels (RNAseq was therefore excluded), Factor 1 was regulated after 6 h reflecting the later effects on target genes and phospho-/proteins, while Factor 4 was regulated after 24 h reflecting the late events on the phospho-/protein level (RNAseq was therefore excluded) (Fig. 2d and supplementary Table 7). Since it is well established that omics approaches are not sensitive enough to capture the fine regulation of transcription factors and early regulated genes,^25^ we conducted a transcription factor enrichment analysis in addition, based on the differentially regulated molecules after 6 h of trametinib 100 nM treatment, identifying a dense network of transcription factors involved in the early time points (i.e. wave 2, 1 h) (Fig. 2e and supplementary Table 7). supplementary Fig. 2c indicates the top 20 most differentially regulated molecules upon treatment in each factor. Taken together, this analysis step identified a variety of genes, proteins and phosphoproteins differentially regulated upon MAPKi for each activation wave and under the control of the MAPK pathway in pLGG. While some molecules were identified in several omics layers (e.g., THBD), a majority of molecules were identified in a single omics layer only, highlighting the complementarity of this multi-omics dataset, and the complexity of the underlying molecular processes. We further filtered this list using the SeneQuest database, in order to only retain the molecules related to senescence processes (*n* = 108 upregulated molecules, and *n* = 81 downregulated molecules, as summarized in supplementary Table 8). We further validated biologically relevant downstream factors in this OIS and SASP signature upon MEKi treatment by RT-qPCR, with *Jun*, *JunB* and *IL6* being all downregulated upon trametinib 24 h treatment (supplementary Fig. 2d and supplementary Table 1).

We finally inferred the relationship between the molecules identified in each waves using the OmniPath database, allowing us to draw a signaling pathway of the MAPK-related genes involved in senescence in our DKFZ-BT66 pLGG cells (Fig. 2f–g). As expected, we found molecules related to OIS (i.e. TP53, CDKN2A/B), the SASP (i.e. IL-1B, IL-6, IL-8, PLAU, MMP-1), and cell survival (BCL2A1, BAD). Molecules from the p38, JAK/STAT and ATM/ATR pathways were also found upregulated in the senescent cells and under the control of the MAPK pathway (Fig. 2f). On the other hand, pathways known to be downregulated in pLGG cells such as cell cycle (i.e., CDKs) and AKT were also found downregulated. In addition, the transcription factor POU5F1, the CK2 kinase and IRS-1 were downregulated in our senescent pLGG cells, alongside molecules involved in cell division (aurora kinases), tubule formation (GSK3B/TAU), and post-translational modification (GFPT-1) (Fig. 2g). In summary, these analyses provide a specific and comprehensive signaling network map of MAPK-OIS-SASP interdependencies in pLGGs.

To control the relevance and specificity of our signatures, we selected 100 random lists of 108 upregulated genes and 100 random lists of downregulated genes from the SeneQuest database prefiltered in our study (supplementary Table 9). In order to test the hypothesis that the pathway connection (i.e., protein–protein interaction network) varies with randomly selected genes, we performed a String analysis to assess the connectivity of each of these random lists, and compared them to our OIS_UP and OIS_DN lists. For the up-regulated lists, our OIS_UP signature topped the ranking, with the network showing the highest number of edges, average node degree and average local clustering coefficient. On the other hand, the OIS_DN signature topped the ranking with the networking showing the highest number of edges and average node degree, and being in the top 5 for the average local cluster coefficient (supplementary Table 10). Taken together, this shows that the degree of connectivity of the molecules identified in our study is higher than random, and highlights the relevance of our results.

### The multi-omics MAPK/OIS/SASP molecular signatures are enriched in primary pLGG and associated with better PFS in pLGG patients

**Fig. 3:**
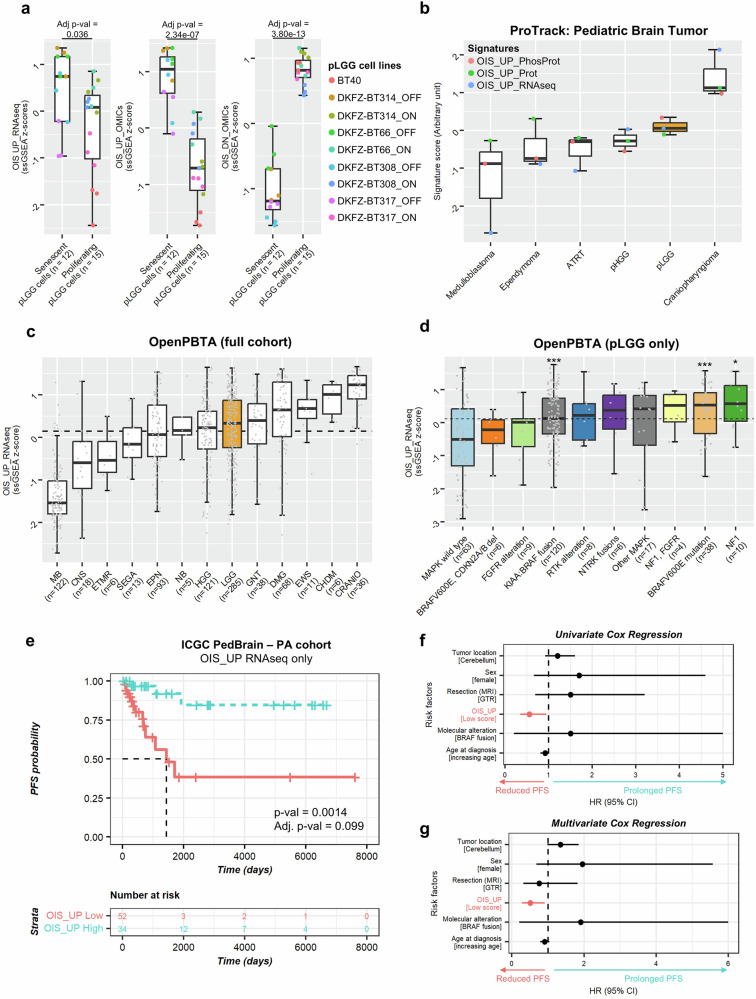
Validation of the MAPK/OIS/SASP molecules in primary pLGG samples. **a** Boxplot depicting the ssGSEA z-score of the OIS_UP or OIS_DN molecules (from RNAseq layer only, or all three omics layers combined) in 9 patient-derived pLGG models (ON = proliferating cells, OFF = senescent cells, BT40 = proliferating). **b** Boxplot depicting the signature score (z-score sum, arbitrary unit) of the OIS_UP molecules from the RNAseq, proteomics and phosphoproteomics layers in *n* = 6 pediatric glioma entities from the ProTrack Pediatric Brain Tumor dataset from Petralia et al. Each dot represents the median OIS_UP z-score of each tumor entity dataset indicated, for all three omics layers from supplementary (**a**–**c**). **c** Boxplot depicting the ssGSEA z-score of the OIS_UP genes from the RNAseq layer in *n* = 13 pediatric glioma entities from the Open Pediatric Brain Tumor Atlas. Dashed line depicts the overall median. MB medulloblastoma, EWS Ewin Sarcoma, EPN ependymoma, NB neuroblastoma, CNS other CNS embryonal tumor, ETMR embryonal tumor with multilayer rosettes, SEGA Subependymal Giant Cell Astrocytoma, CHDM chordoma, HGG high-grade glioma, CRANIO craniopharyngioma, GNT glial neuronal tumor, DMG diffuse midline glioma, pLGG low-grade glioma. **d** Boxplot depicting the ssGSEA z-score of the OIS_UP genes from the RNAseq layer in *n* = 10 pediatric low-grade glioma molecular subgroups from the Open Pediatric Brain Tumor Atlas. Dashed line depicts the overall median. Boxplots depict the median, first and third quartiles. Whiskers extend from the hinge to the largest/smallest value no further than 1.5 * IQR from the hinge (where IQR is the inter-quartile range). Significance was calculated with one-way ANOVA followed by the Tukey’s ‘Honest Significant Difference’ test. Depicted significance is in comparison to the group “MAPK wild type”, all other comparisons to other groups were not significant. **e** Kaplan–Meier curve of primary BRAF-driven PA samples from the ICGC PedBrain cohort grouped based on their enrichment for the OIS_UP genes from the RNAseq layer (*p*-value from log-rank test, and adjusted *p*-value corrected by Bonferroni method after multiple testing to identify the optimal cut-off with the best raw *p*-value). **f** Forest plot depicting the hazard ratio (HR) calculated in a univariate Cox proportional hazards model evaluating which clinico-molecular features are significantly associated with PFS. **g** Forest plot depicting the HR calculated in a multivariate Cox proportional hazards model evaluating independent prognostic factors

Gene expression datasets from other pLGG-derived cell lines were used to validate the identified MAPK-dependent OIS/SASP molecules, using only the genes upregulated, or all omics molecules up- and downregulated as gene signatures (supplementary Table 8). Both the significant enrichment of the upregulated molecules as well as the depletion of downregulated molecules in the senescent cells compared to their proliferative counterpart was validated (Fig. 3a and supplementary Table 11).

In primary samples from the ProTrack Pediatric Brain Tumor dataset,^26^ the upregulated MAPK-dependent OIS/SASP genes, proteins, and phosphoproteins were upregulated in pLGG compared to almost all the other brain tumor entities, except for craniopharyngioma, known to also have some OIS components.^27^ (Fig. 3c, supplementary Fig. 3a–c and supplementary Table 12). In the Open Pediatric Brain Tumor Atlas (OPBTA),^28^ the upregulated MAPK-dependent OIS/SASP genes were enriched in pLGG (Fig. 3c and supplementary Table 13). Interestingly, 4/5 entities with a high signature score (glial neuronal tumor [GNT], diffuse midline glioma [DMG], chondroma [CHDM], craniopharyngioma [CRANIO]) were also found to have an increased MAPKi sensitivity score (MSS) in another study,^29^ validating that our signature retained components related to MAPK pathway activity. When split by MAPK alteration, 7/8 pLGG subtypes with a MAPK alteration had an enrichment of MAPK-dependent OIS/SASP genes above the overall median, while pLGG subtypes with a wild type MAPK pathway, or pLGG with a BRAF^V600E^ mutation and *CDKN2A/B* deletion (known to be more aggressive) were below the overall median (Fig. 3d and supplementary Table 13). The pLGG subgroups with an NF1 alteration, BRAF^V600E^ mutation or a KIAA1549::BRAF fusion had a significant enrichment for the MAPK/OIS/SASP related genes compared to the MAPK wild type (Fig. 3d).

Finally, the BRAF-driven PA patients with an enrichment of MAPK-related OIS/SASP genes (Fig. 3e) or molecules (all omics molecules used as a gene signature, supplementary Fig. 3d and supplementary Table 14) had a prolonged progression-free survival (PFS), in line with the concept that tumors in OIS are less at risk of progressing. Univariate Cox regression analysis evaluating which individual factors were associated with PFS showed that only the OIS_UP signature (HR: 0.23, 95%CI: 0.08–0.65, *p* = 0.0058) and extent of resection (HR: 2.1, 95%CI: 1.1–4, *p* = 0.019) were significantly associated with PFS (Fig. 3f). Multivariate Cox regression analysis using all predictors confirmed that the OIS_UP signature was the only independent prognostic factor (HR: 0.25, 95%CI: 0.084–0.77, *p* = 0.016) (Fig. 3g). The same trend was observed in the independent dataset from the OPBTA, without reaching significance (supplementary Fig. 3e and supplementary Table 14).

Of note, the OIS_UP signature z-scores showed similarity with no significant (*p*-value) differences across pLGG histological subtypes, suggesting a potential applicability across all pLGG subtypes (supplementary Fig. 3f).

Taken together, these data suggest that the MAPK-related OIS/SASP molecules identified here are relevant to the biology and clinical course of primary pLGG, thereby validating our approach and results.

### ssGSEA analysis identifies (co-) regulated pathways in pLGG that could be targeted in first line or in combination with MAPK inhibitors

**Fig. 4:**
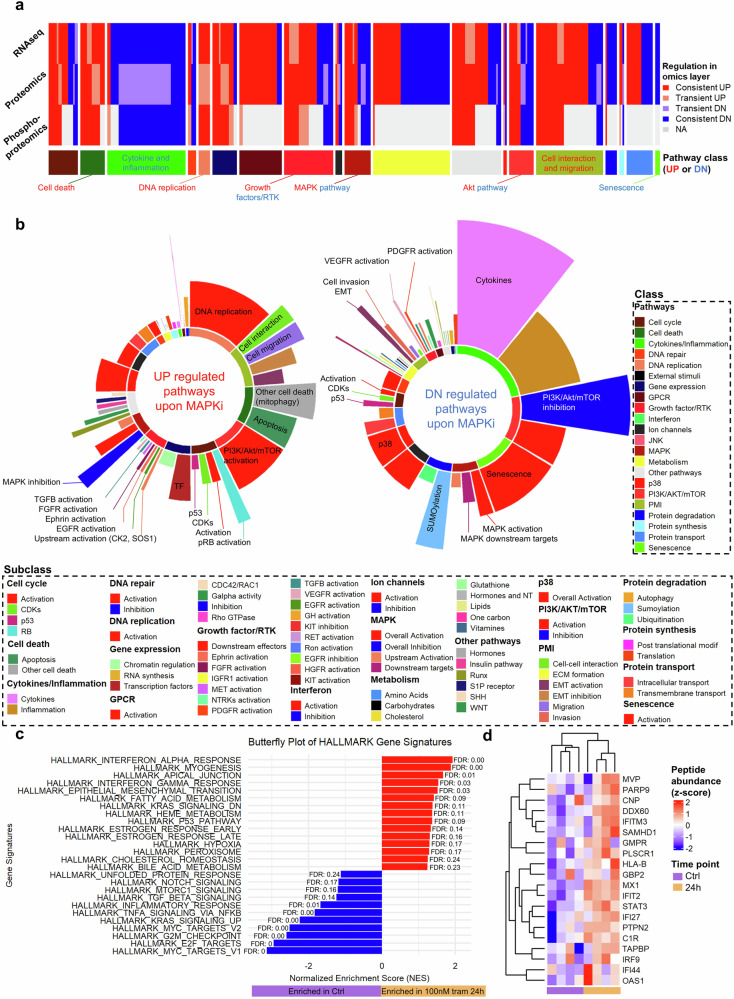
Identification of new co-regulated pathways in MAPKi treated pLGG cells. **a** Heatmap depicting the filtered signatures considered to be consistently regulated upon MAPKi treatment (trametinib 100 nM) in the senescent DKFZ-BT66 cells through omics layers. RNAseq = signature from RNAseq dataset, Prot = signatures from proteomics dataset. PhosProt = signatures from phosphoproteomics dataset. **b** Sunburst chart summarizing the signaling pathways upregulated (UP) and downregulated (DN) upon MAPKi (trametinib 100 nM) in the senescent DKFZ-BT66 cells. The inner circle represents the proportion (sections’ length) of signatures belonging to a given category relative to the total amount of signatures belonging to that category, while the outer circle represents the proportion (bars’ height) of signatures belonging to a given sub-category relative to the total amount of signatures belonging to that sub-category. **c** Butterfly plot depicting the results from the GSEA comparing RNAseq expression of the 100 nM trametinib 24 h samples and control, using the HALLMARK gene set. Only the signatures with an FDR q-val < 0.25 are depicted. NES normalized enrichment score, FDR false discovery rate. **d** Heatmap depicting the scaled peptide abundance of top 20 most upregulated proteins related to interferon activity and participating in the core enrichment of the HALLMARK signatures related to interferon alpha and gamma in the RNAseq data from (**c**)

To go beyond MAPK-related molecules only related to OIS and SASP processes, we broadened our approach and investigated all other pathways differentially regulated upon MAPK inhibition on the gene, protein, and phosphoprotein level in our pLGG cell line (supplementary Figs. 4–6 and supplementary Table 15). The omic-specific signatures, manually classified into main classes, were used to measure enrichment scores via ssGSEA, and their overall regulation pattern through time was investigated (supplementary Fig. 7a). Only the signatures that showed a consistent regulation through all omics layers, reflecting robust regulation, were kept for further analysis (Fig. 4a, supplementary Fig. 7b and supplementary Table 16).

Among the pathways upregulated upon MAPK inhibition, we identified signatures related to DNA replication, cell interaction and migration, mitophagy and apoptosis, Akt, MAPK upstream activators (SOS1, CK2) and other RTK-related pathways. On the other hand, we identified signatures related to cytokines, senescence, MAPK activity and Akt pathway inhibition as being downregulated, as expected, upon MAPK inhibition (Fig. 4b and supplementary Table 17).

We finally performed an end-point analysis (24 h treatment vs control) in a GSEA analysis (RNAseq data only), and found that the most consistently upregulated pathways upon MAPKi treatment were related to interferons activity (Fig. 4c and supplementary Table 18). Several genes participating in the core enrichment of the interferon signatures were also enriched upon 24 h treatment on the protein level (Fig. 4d).

This comprehensive dataset of MAPK-related co-regulated pathways in pLGG provides a valuable resource and highlights potential novel targets to be further investigated, either as single agents or in combination, for the treatment of pLGG.

### Selected inhibitors targeting co-regulated pathways highlight new vulnerabilities in pLGG

**Fig. 5:**
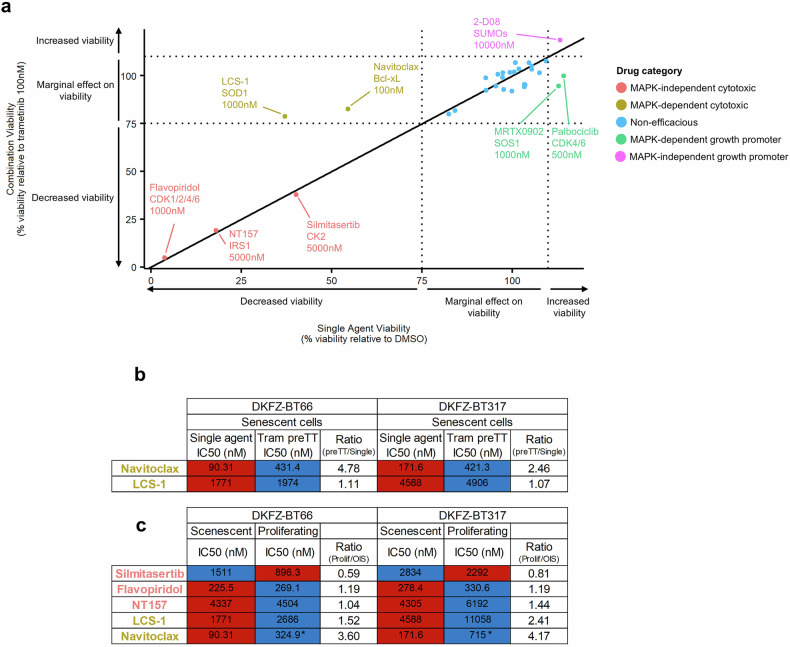
Drug screen validation of key drugs worth investigation as first line or in combination with MAPKi in pLGG cells. **a** Summary dotplot depicting the results from a single-dose drug screen using *n* = 35 clinically relevant drugs targeting newly identified molecules and pathways in the senescent DKFZ-BT66 cells. Relative metabolic activity compared to the corresponding control is shown. The drug screen was performed in technical duplicates, and a single biological replicate. **b** Summary heatmap showing the IC_50_ values (nM) of a given MAPK-dependent cytotoxic drug either used as a single agent or after a 24 h pre-treatment with 100 nM trametinib in senescent DKFZ-BT66 and senescent DKFZ-BT317 cells. The data depict the average IC_50_ from *n* = 3 independent biological replicates. **c** Summary heatmap showing the IC_50_ values (nM) of a given MAPK-(in)dependent cytotoxic drug in senescent or proliferating DKFZ-BT66 and DKFZ-BT317 cells. The data depict the average IC_50_ from *n* = 3 independent biological replicates. * IC_50_ taken from historical experiments conducted in the same conditions, published by Sigaud et al. for the DKFZ-BT66,^21^ and Selt et al. for the DKFZ-BT317.^53^

A panel of 35 clinically relevant drugs was assembled to target key genes, proteins and phosphoproteins (Fig. 2f, g) and pathways (Fig. 4b) identified in our multi-omics analysis (supplementary Table 19). A single dose drug screen was carried out in the senescent DKFZ-BT66 cells at a biologically/clinically relevant dose (close to known in vitro IC_50_ and/or around the Cmax concentration observed in patients, respectively), either alone or after a 24 h pre-treatment with trametinib (100 nM) (Fig. 5a and supplementary Table 20). Aside from drugs that remained ineffective (marginal effect) at the tested concentration, either alone or in combination, four potential classes of effective drugs were identified: *n* = 1 MAPK-independent growth-promoting drug (2-D08), increasing the metabolic activity regardless of the treatment modality; *n* = 2 MAPK-dependent growth-promoting drugs (MRTX0902 and palbociclib), increasing metabolic activity only when not combined with MAPKi; *n* = 2 MAPK-dependent cytotoxic drugs (LCS-1 and navitoclax, as expected); *n* = 3 MAPK-independent cytotoxic drugs (flavopiridol, NT157 and silmitasertib) (Fig. 5a).

While cell count experiments did not validate the growth promoting effects of the first two classes defined above (supplementary Fig. 8a–c), we could validate the reduction of metabolic activity of the latter two drug classes, also in the KIAA1549::BRAF fusion pLGG model DKFZ-BT317 (Fig. 5b, c and supplementary Table 5). Navitoclax and LCS-1 IC_50_ values were on average 3.62-fold and 1.09-fold lower in the single agent treated compared to the trametinib pre-treated senescent pLGG cells, respectively (Fig. 5b and supplementary Fig 1e, 8d). Interestingly, both drugs’ IC_50_ values were 3.89-fold and 1.97-fold lower in the senescent pLGG cells compared to their proliferative counterpart, respectively (Fig. 5c and supplementary Fig 1e, 8e). Flavopiridol and NT157 IC_50_ values were 1.19-fold and 1.24-fold lower in the senescent pLGG cells, respectively, while silmitasertib IC_50_ values was 1.47-fold lower in the proliferating cells (Fig. 5c and supplementary Fig. 8f–h).

Importantly, target expression for (1) navitoclax was derived from the multi-omics integration (all layers), (2) LCS-1 was derived from the proteomics dataset, (3) NT157 was derived from both proteomics and phosphoproteomics, and (4) flavopiridol and silmitasertib were derived from kinase-substrate enrichment analysis using the phosphoproteomics dataset, highlighting the complementarity of the multi-omics approach.

**Fig. 6:**
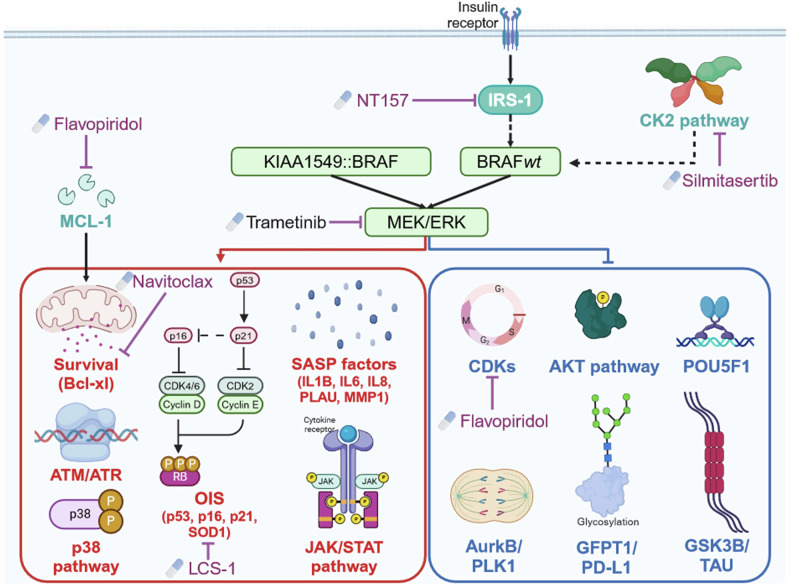
The MAPK/OIS/SASP axis in pLGG cells. Graphical depiction of the validated signaling pathway and molecules in the senescent pLGG models. Created in BioRender. Sigaud, R. (2024) https://BioRender.com/j47h916

Taken together, we have identified MAPK-(in)dependent effectors that could represent new vulnerabilities in senescent pLGG and are worth further pre-clinical evaluation to assess their true potential for the treatment of pLGG patients (Fig. 6).

## Discussion

Our study presents the first comprehensive multi-omics dataset of a patient-derived pLGG cell line under MAPK inhibition, encompassing RNA sequencing, proteomics, and phosphoproteomics at various time points. This unprecedented dataset offers an integrated view of the molecular response to MAPKi treatment, providing insights into the dynamic interplay between transcriptional, translational, and post-translational regulatory mechanisms in pLGGs. The integration of these multiple layers of biological information allowed for a more nuanced understanding of the signaling cascades and compensatory mechanisms activated in response to MAPK inhibition. It has provided novel insights into the molecular mechanisms underlying pLGG biology, revealed the individual players of the complex interplay between MAPK signaling, OIS, and the SASP, and identified potential therapeutic strategies.

Our data demonstrate a time-dependent inhibition of MAPK, OIS, and SASP upon trametinib treatment, suggesting a strong connection between MAPK pathway activity and these processes in pLGG cells. This finding is consistent with previous studies that have implicated MAPK signaling in senescence induction and maintenance in pLGG artificial models.^12,20^ However, our study is the first to comprehensively map this relationship in pLGG using a multi-omics approach. Indeed, the OIS-related mRNA, protein, and phosphoprotein levels were decreased upon MAPKi treatment, with a trend for the protein/phosphoprotein levels to revert to baseline after 24 h—likely reflecting the lesser robustness of these protein-based signatures compared to the mRNA-based one. We therefore validated OIS inhibition functionally by testing sensitivity to OIS-specific senolytic drugs in combination with MAPKi. Our results showed that MAPK inhibition induces resistance to senolytic agents such as navitoclax. A 24 h pre-treatment with trametinib, time necessary to decrease the OIS/SASP molecular programs, led to a resistant of the senescent pLGG cell line to navitoclax and A-1331852. Interestingly, a co-treatment didn’t induce this resistance, most likely because the OIS/SASP molecules were still engaged in the senescence process during the co-treatment, resulting in a sensitivity similar to the non-MAPKi treated cells. This observation has significant clinical implications, as it suggests that combining MAPK inhibitors with senolytics may need to be carefully scheduled regarding sequence and timing of the individual drugs. This finding contrasts with some previous studies in other cancer types, where combinations of targeted therapies and senolytics have shown promise.^30,31^ The discrepancy in outcomes can be attributed to the unique biology of pLGGs. pLGGs are typically driven by a single oncogenic event and maintain a relatively benign biology. In these tumors, the OIS failsafe mechanism functions effectively, acting as a natural tumor suppressor. In that context, MAPK inhibition interferes with this protective OIS mechanism, making the cells less susceptible to senolytic agents. This scenario differs markedly from what occurs in higher-grade, more aggressive cancers. In those cases, targeted therapies often induce therapy-induced senescence (TIS), rendering the cancer cells more vulnerable to senolytic agents, which can then push the cells over the apoptotic threshold. It was indeed observed that, in combination therapies conducted in MAPK-driven tumor models (pleomorphic xanthoastrocytoma, colorectal cancer) in a proliferating state, MAPKi treatment led to TIS, thereby sensitizing the cells to senolytics.^30,31^ Our results highlight the need for careful consideration of treatment schedule in pLGG and underscores the importance of considering the cellular state, genetic makeup and genomic stability when designing treatment strategies.

The enrichment of our identified MAPK-dependent OIS/SASP signatures in primary pLGG samples and their association with better progression-free survival validate the clinical relevance of our findings, even when taking into account tumor location, level of resection, and genetic alteration. This not only supports the biological significance of our in vitro model, but also suggests that these signatures could potentially serve as prognostic markers in pLGG patients. This in line with previous work showing that patients with an enrichment for senescence-associated signatures had a prolonged PFS.^13^ The use of such gene-based signatures, while not routine established yet in clinical pipelines, may have significant potential in the future, as full molecular characterization becomes more widely available as illustrated by the LOGGIC Core.^32^ For instance, in patients with high OIS_UP expression, it may be worthwhile to investigate whether extended MRI intervals during follow-up (e.g., less frequent scans) could be as safe as current protocols, as these patients tend to have a very low risk of progression. Future studies should focus on validating these signatures in larger, prospective cohorts. Furthermore, we validated on a single-gene level biologically relevant downstream factors belonging to our OIS_UP signature upon MEKi treatment. In particular, the downregulation of proteins from the AP-1 complex (i.e., c-Fos, jun, junB) and SASP factors (IL-1B and IL-6) supports the biological relevance of our integrative signature, and suggests AP-1 complex inhibition as a mechanistic driver of SASP attenuation in the pLGG MAPK-OIS axis. This is in line with several evidences that AP-1 plays a key role in senescence and SASP factor regulation in other tumor types.^33,34^

The identification of molecules related to p38, JAK/STAT, and ATM/ATR pathways as being upregulated and under MAPK control in senescent pLGG cells is particularly intriguing. These pathways have been implicated in senescence in other contexts,^35–37^ but their specific roles in pLGG are unclear. On the other hand, we observed a downregulation upon MAPK inhibition of effectors related to senescence but never observed before in a pLGG background. For instance, POU5F1, coding the Oct4 transcription factor involved in the self-renewal of stem cells and capable of reverting senescence,^38^ was found downregulated in our pLGG model. In addition, we found evidence of a downregulation upon MAPK inhibition of the Glutamine--Fructose-6-Phosphate Transaminase 1 (GFPT-1, aka GFAT1), involved in the glycosylation of many protein. In particular, GFPT1 reduced activity was associated with a destabilization of the PD-L1 protein in lung cancer, limiting the effects of immunotherapies.^39^ This is line with the extremely low expression level of PD-L1 at the surface of pLGG tumor cells,^40^ and the fact that MAPK activity can promote an immunosuppressive microenvironment via a downregulation of PD-1/PD-L1 molecules, as observed in colorectal cancer or melanoma.^41,42^ This suggest that such a combination of MAPKi and ICI could be promising in pLGG and warrants further evaluation.

Our drug screening results have revealed several promising therapeutic strategies: MAPK-independent cytotoxic drugs (flavopiridol, NT157, and silmitasertib) showed efficacy regardless of MAPK inhibition, suggesting their potential as single agents or in combination with MAPK inhibitors. For instance, the cyclin-dependent kinase inhibitor flavopiridol has been shown to downregulate MCL-1,^43^ hence conferring increased sensitivity in combination with senolytics in a model of senescent triple-negative breast cancer.^44^ Interestingly, flavopiridol also inhibited pLGG cells in their proliferation phenotype, probably via CDK inhibition. This indicates a potential double-edged sword effect, with this drug potentially capable of targeting both proliferating and senescent pLGG tumor cells.

While NT157 is no longer in clinical development, its target IRS-1 has been long reported as a key mediator of brain development, where its expression is high at early stages of development, and then decreases with age.^45^ In particular, its overexpression promoted cell proliferation in cholangiocarcinoma,^46^ while its knockdown reduced cell proliferation and downregulated both Akt and MAPK pathways in BCR-ABL cells in vitro.^47^ In our model, IRS-1 expression is reduced by the MAPK pathway, hence reducing its positive effects on cell proliferation but possibly retaining its effects on cell survival, potentially explaining the decreased viability observed upon NT157 treatment in our senescent pLGG cells. The exact mechanism governing this sensitivity warrants further evaluation, while testing of the other IRS-1 inhibitor NT219, which recently showed promises in combination with MAPKi,^48^ could therefore represent another interesting modality to further test in pLGG preclinical studies.

Silmitasertib is a CK2 inhibitor that has received a rare pediatric disease designation (RPDD) from the US FDA for the treatment of medulloblastoma, and more recently neuroblastoma, and will soon be investigated in a Phase 1/2 trial for the treatment of pediatric and adult relapsed/refractory solid tumors (NCT06541262). With IC_50_ in a clinically achievable nanomolar range in both proliferating and senescent pLGG cells, silmitasertib also holds promises for the treatment of another pediatric disease.

OIS-specific vulnerabilities were identified, with navitoclax and LCS-1 showing lower IC_50_ values in senescent cells compared to proliferating cells, and higher IC_50_ when the cells were pre-treated with the MEKi trametinib. While we have already discussed navitoclax earlier, LCS-1 represents another potential senolytic-like agent for pLGG. LCS-1 inhibits Superoxide dismutase 1 (SOD1), an antioxidant responsible for neutralizing supercharged oxygen radicals in the cell and involved in the induction of senescence, notably via a mechanism involving p16.^49^ While other studies have shown that SOD1^−/^^−^ mice accumulated senescent cells triggered by the accumulation of reactive oxygen species (ROS),^50^ it appears that in pLGG cells, SOD1 protein expression is under the control of the MAPK pathway, and involved in the control of OIS. While LCS-1 is a tool compound not in clinical development, the antisense oligonucleotide tofersen, which can mediate the degradation of SOD1 mRNA to reduce SOD1 protein synthesis, is now FDA approved for the treatment of amyotrophic lateral sclerosis.^51^ These findings highlight the complementarity of the multi-omics approach, as target expression for each drugs was derived from at least one of each omics dataset, and provide a rationale for further pre-clinical and clinical investigation of these compounds in pLGG.

While our study provides valuable insights, it has some limitations. A first limitation is the generation of the initial multi-omics dataset in a single cell line only, due to resource restriction of limited true pLGG cell lines available at the time to generate the large amount of protein needed to start the analyses. While the number of cell lines for the initial proteomic data set is low with an *n* = 1, this data served as a discovery basis only, and results were always validated in multiple true pLGG cell lines and/or bona fide pLGG patient-derived samples, compensating for the limitation in number of cell lines used for the initial proteomic analysis. Another limitation is the focus on in vitro models, which may not fully recapitulate the complexity of the tumor microenvironment. In particular, the identification of interferon pathways up-regulated upon MAPKi treatment suggest an indirect (paracrine) role of MAPKi on the tumor microenvironment, and a potential applicability for immunotherapies in combination with MAPKi in pLGG. This is in line with previous studies suggesting that MAPK pathway activity promotes an immune evasive environment, that can be reversed upon MAPKi, notably associated with the increased activation of interferons and antigen-presenting pathways in BRAF^V600E^-driven melanoma.^52^ Such combinations of MAPKi and immune modulators have already shown early promises, for instance in MAPK-driven colorectal cancer.^42^ Future studies should validate these results in more advanced models, such as GEMMs, patient-derived xenografts or organoids. Finally, while we have identified several potential therapeutic targets, their efficacy and safety in vivo remain to be established. Comprehensive pre-clinical evaluation of these targets, alone and in combination, will be crucial to validate their therapeutic potential. The mechanisms underlying the differential drug responses in senescent versus proliferating cells warrant further investigation. Understanding these mechanisms could lead to more refined treatment strategies and potentially improve the outcome observed with current therapies.

Nonetheless, this dataset represents a useful resource for the scientific community, serving as a reference point for future studies in pLGG, and on the MAPK in general. pLGG being a single pathway disease, our dataset represents a unique resource to study MAPK pathway activity upon MAPKi without the interreferences of other mutations, often co-occurring in other entities. Researchers can use this data to generate and test new hypotheses, validate findings in different experimental settings, or apply advanced bioinformatics approaches to explore potential therapeutic targets or biomarkers. The time-resolved nature of our dataset enables the study of temporal dynamics in response to MAPKi, which could help elucidate the compensatory mechanisms activated during MAPK pathway inhibition, in an approach not necessarily OIS-centered. We hope this dataset will facilitate collaborative efforts and contribute to ongoing research in pLGGs, potentially aiding in the development of more effective treatment approaches for these challenging pediatric tumors.

In conclusion, this study has uncovered novel insights into OIS in pLGG, and its dependency towards MAPK pathway. We identified MAPK-dependent OIS molecular signatures and potential therapeutic targets in pLGG, paving the way for more effective and personalized treatment approaches for this challenging pediatric brain tumor. Future research should focus on validating these findings in pre-clinical settings and exploring combination strategies that exploit the unique vulnerabilities of senescent pLGG cells.

## Materials and methods

### Cell lines and drug treatments

Pilocytic astrocytoma cell lines (DKFZ-BT66, DKFZ-BT317) established in our previous studies^13,53^ were cultured in Astrocyte Basal Medium (ABM, cat. no. CC-3187, Lonza) and supplement (FBS 3% v/v, L-Glutamine 1% v/v, GA-1000 0.1% v/v, Ascorbic acid 0.1% v/v, HEGF 0.1% v/v, insulin 0.25% v/v, from Astrocyte Growth Medium BulletKit, cat.no. CC-3186, Lonza) and 1 µg/ml doxycycline (cat. no. sc-337691, Santa Cruz) in order to induce cell proliferation, as described previously.^13,53^ All cells were cultured under 5% CO_2_ atmosphere, at 37 °C. Cell lines were authenticated using Multiplex Cell Authentication,^54^ and purity was validated using the Multiplex cell Contamination Test,^55^ both performed by Multiplexion (Heidelberg, Germany). Seeding densities can be found in supplementary Table 21.

Cells were treated with 100 nM trametinib at the indicated time points before sample collection. For the BCL-XLi combination dose-response curves, cells were treated with a combination of 100 nM trametinib and varying concentrations of the BCL-XLi navitoclax or A-1331852, as indicated in supplementary Fig. 1d (see supplementary Table 22 for drugs details). A drug screen was performed using the drug library found in supplementary Table 19. For both experiments, metabolic activity (Cell Titer Glo) measurement was carried out 72 h after treatment (+/− 24 h trametinib 100 nM pre-treatment, as indicated) following the manufacturer’s instructions using a FLUOstar OPTIMA automated plate reader (BMG Labtech).

### Western blot analysis

At the indicated time point, cells were washed in PBS and lysed in SDS-Buffer containing PhosSTOP phosphatase inhibitors (cat. no. 49068450001, Sigma Aldrich) and cOmpleteTM mini proteinase inhibitors (cat. no. 11836153001, Sigma Aldrich). Protein concentration was measured with the PierceTM BCA Protein Assay Kit (cat. no. 23227, ThermoFisher Scientific) using the FLUOstar OPTIMA automated plate reader (BMG Labtech) to prepare 15 µg/ml samples. Gel electrophoreses was performed using 10% acrylamide gels. Proteins were transferred to a PVDF membrane using the Trans-Blot Turbo RTA Mini 0.45 μM LF PVDF Transfer Kit (cat. no. 1704274, Biorad) with the Trans-Blot Turbo Transfer System (Biorad). Immunodetection was done with Amersham ECL Prime Western Blotting Detection Reagent (cat. no. RPN2232, GE Healthcare Dharmacon) using the Azure c400 imaging system (Azure Biosystems). Quantification was done using ImageJ (v2.9.0). The antibodies used and further details are listed in supplementary Table 23. Uncropped images can be found in the Supplementary Materials.

### RNA extraction and qPCR

At the indicated time point, RNA extraction was performed using the RNeasy Mini Kit (cat. no. 74104, Qiagen) with on-column DNase digestion according to the manufacturer’s instructions. cDNA synthesis was done using the RevertAid First Strand cDNA Synthesis Kit (cat. no. K1622, ThermoFisher Scientific) following the manufacturer’s protocol. qPCR was performed using an ABI 7500 Real Time PCR cycler (Applied Biosystems) with ABI 7500 Software v2.3 (Applied Biosystems) and qPCR Mastermix for SYBR® Green I (cat. no. 4309155, ThermoFisher Scientific). The ΔΔCt method was used to perform relative quantification. ACTB and TBP were used as housekeeping genes for all in vitro samples. The list of primers and sequences used can be found in supplementary Table 23.

### RNA sequencing on pLGG cell lines

For RNA sequencing, RNA samples for each time point from 3 biological replicates were processed to remove rRNA using QIAseq FastSelect (Human/Mouse/Rat 96rxns). cDNA synthesis was performed using the NEBNext RNA First Strand Synthesis and NEBNext Ultra Directional RNA Second Strand Synthesis Modules (New England BioLabs). Library preparation was performed using the NEBNext Ultra II DNA Library Prep Kit for Illumina (New England BioLabs), and 2 × 100 bp paired-end sequencing (60x sequencing depth) was performed on an Illumina NovaSeq 6000 sequencing platform. No material was left after extraction. The generated Fastq files were submitted to the RNAseqWorkflow pipeline on the One Touch Pipeline (OTP) platform to proceed with automated reads trimming, mapping to the hg38/GRCh38.p13 human genome, annotation to ENSEMBL version 84, and calculation of TPM per transcript.^56^

### LC-MS/MS phospho-/proteomics analysis of pLGG cell line

For phospho-/-proteomics, 50 mg of cells were harvested for each time point from four biological replicates. Cells were lysed and homogenized in urea buffer (30 mM Tris Base 1 M, 7 M Urea, 2 M Thiourea, adjusted to pH 8.5) with a TissueLyser (Qiagen) and supernatants were collected after centrifugation. Protein concentration was determined by means of Pierce 660 nm Protein Assay (Thermo Fischer Scientific). For LC-MS analysis a modified magnetic bead-based sample preparation protocol according to Hughes and colleagues was applied.^57^ A first enrichment of phosphopeptides was performed using an IMAC approach, followed by a second enrichment step was performed using TiO_2_. For the LC-MS acquisition an Orbitrap Fusion Lumos Tribrid Mass Spectrometer (Thermo Fisher Scientific) coupled to an Ultimate 3000 Rapid Separation liquid chromatography system (Thermo Fisher Scientific, Idstein, Germany) equipped with an Acclaim PepMap 100 C18 column (75 µm inner diameter, 25 cm length, 2 µm particle size from Thermo Fisher Scientific) as separation column and an Acclaim PepMap 100 C18 column (75 µm inner diameter, 2 cm length, 3 µm particle size from Thermo Fisher Scientific) as trap column were used. Data analysis was performed with Proteome Discoverer (version 2.4.1.15, Thermo Fisher Scientific).

### Method for the Functional Integration of Spatial and Temporal Omics data (MEFISTO) analysis

A data integration from the RNAseq, proteomics, and phosphoproteomics datasets, taking into account the time component, was performed using the Method for the Functional Integration of Spatial and Temporal Omics data (MEFISTO) (MOFA2 package – version 1.6.0).^22^ Read-counts from RNAseq were normalized as recommended by the MEFISTO creators, by applying size factor normalization from the DESeq2 package (version 1.36.0), variance stabilizing transformation using the iDEP2.0 web application,^58^ and batch corrected with the limma package (version 3.52.4). Peptides (supplementary Table 24) and phosphopeptides abundances (supplementary Table 25) were normalized and batch-corrected using the PhosR package^59^ (version 1.6.0) following the authors’ instructions (see also code deposited in GitHub in “Statistics and Reproducibility section”). Missing values were imputed with PhosR using a Site- and condition-specific imputation followed by a Paired tail-based imputation. The normalized datasets (Dataset 1) were then filtered, as recommended by the authors. For the RNAseq dataset, only protein coding genes with at least one treatment time point with a log2FC > 1 or <-1 and a *p* < 0.05 were kept. For the proteomics and phosphoproteomics, phospho-/peptides with a log2FC > 1 or < -1 in at least one treatment time point were kept. The filtered datasets (supplementary Table 6) were finally used to perform the MEFISTO analysis, using the default settings recommended by the authors. The data integration was done using scaled data, in order to standardize the multi-omics dataset. Unsupervised hierarchical clustering was performed as quality control, to ensure that the harmonized datasets captured the temporal regulation of MAPKi. Several iterations for the model fitting were executed, with up to *n* = 15 factors (default setting of MEFISTO). The percentage of variance explained by each factor for each omics layers was collected, and scaled as z-scores (supplementary Table 26). Iterations until *n* = 5 factors gave factors explaining a significant degree of the variance (i.e., z-score > 0). All other iterations with *n* > 5 gave additional factors that did not explain a significant amount of the variance in each omics layer. We therefore selected the model with *n* = 5 factors for further analysis. Significant genes, proteins and phosphopeptides were selected if their weight z-score was >1 or < −1. All identified molecules can be found in supplementary Table 7.

### Transcription factor enrichment analysis (TFEA)

TFEA was performed using the iDEP2.0 web application (using the TF: ENCODE reference), and using the OmniPath database to infer putative transcription factors associated with the differential expression of the direct downstream genes/proteins from wave 3 (6 h). All transcription factors (TF) can also be found in supplementary Table 7.

### Kinase-substrate enrichment analysis (KSEA)

KSEA was performed using the KSEA App (https://casecpb.shinyapps.io/ksea/) using the PhosphoSitePlus and NetworkIN databases, with a *p*-value cut off of 0.05, and a substrate count cut-off of 5 on the phosphoproteomics dataset. All kinases identified can also be found in supplementary Table 7.

### Mapping of the MAPK/OIS/SASP pathway

Genes, proteins and peptides identified in the MEFISTO analysis were then filtered using the SeneQuest database.^23^ The list of genes from the SeneQuest database was filtered to only retain those with at least three publications supporting a link with senescence. Each gene was then assigned a regulation direction (upregulated or downregulated) based on the one that had the highest number of supporting publications. The filtered list of senescence genes and their regulation can be found in supplementary Table 9. Interactions between the identified molecules were inferred from the OmniPath database.^24^ Interactions were drawn between molecules from the same identified MAPK wave, or between molecules from distinct MAPK waves. Interactions were made based on prior evidence in more than three publications. Phosphopeptides were also used to infer activity of their direct upstream kinases based on the OmniPath database. The complete list of kinases identified can also be found in supplementary Table 7.

### ssGSEA using mRNA-/Protein-/Phosphopeptide-based signatures

For single-sample gene set enrichment analyses, the ssGSEA module from the GenePattern web platform was used.^60^ The collections of signatures ‘Canonical pathways” and “Hallmark gene sets” from the Molecular Signatures Database (MSigDB) were used, and manually annotated into main classes and subclasses. For genes and proteins, gene signatures from the MSigDB were used. For phosphopeptides, gene signatures from the PTM signatures database (PTMSigDB) were used. All annotated signatures can be found in supplementary Table 27.

### Statistics and reproducibility

All statistical analyses were performed in R Studio (version 2023.12.0, with R Version 4.2.1). For signatures overlap and Venn diagrams the “VennDiagram” package (v1.7.3) was used. Significance between groups was calculated using one-way ANOVA followed by the Tukey’s ‘Honest Significant Difference’ test with the “stats” package in R. Significance between groups calculated using one-way ANOVA followed by Dunnett’s multiple comparisons test was done in GraphPad Prism 5 software (Version 5.01, GraphPad Software Inc., San Diego, USA). Longitudinal k-means clustering, an adaptation of the k-means clustering method, was performed using the package “kml” (version 2.4.6). For the correlation plots, waterfall plots and boxplots, the “ggplot2” package (v3.4.1) was used. For heatmaps, the “ComplexHeatmap” package (v2.12.1) was used. Alluvial plots were generated with the “ggalluvial” package (version 0.12.5), and sunburst chart with the “sunburstR” package (version2.1.8). Protein–protein interaction network of transcription factors was depicted using Cytoscape (version 3.10.1).

PFS and Kaplan–Meier curve analyses were carried on using the “Kaplan–Meier analysis using custom data” of the “Survival” module from the R2 online database (https://hgserver1.amc.nl/cgi-bin/r2/main.cgi?option=kaplan_main) using the “scan” method to identify the optimal cut-off, and Bonferonni post-hoc correction to adjust the *p*-value. Univariate and multivariate Cox regression analyses was performed using the “coxph” function from the “survival” package.

To measure ssGSEA scores, the ssGSEA module (v10.1.0) from Gene Pattern was used,^60^ using the recommended parameters instructed in the documentation. ssGSEA scores were not normalized, and considered to be in arbitrary units. For normal GSEA, the GSEA software from the Broad Institute (version 4.0.3) was used.

GraphPad Prism 5 software (Version 5.01, GraphPad Software Inc., San Diego, USA) was used to calculate all IC_50_, using a 4-parameter dose-response model.

All data acquired experimentally were always collected in at least three independent biological replicates. All replicates generated are depicted in the manuscript. Cells were randomly allocated into control and experimental groups for all in vitro experiments. The investigators were not blinded to allocation during experiments and outcome assessment. Sex and/or gender was not a criterion for the study design or data interpretation.

The code used in this manuscript has been uploaded and deposited on GitHub (https://github.com/AGSigaud/OMICs-in-pLGG).

## Supplementary information

Supplementary Table 1

Supplementary Table 2

Supplementary Table 3

Supplementary Table 4

Supplementary Table 5

Supplementary Table 6

Supplementary Table 7

Supplementary Table 8

Supplementary Table 9

Supplementary Table 10

Supplementary Table 11

Supplementary Table 12

Supplementary Table 13

Supplementary Table 14

Supplementary Table 15

Supplementary Table 16

Supplementary Table 17

Supplementary Table 18

Supplementary Table 19

Supplementary Table 20

Supplementary Table 21

Supplementary Table 22

Supplementary Table 23

Supplementary Table 24

Supplementary Table 25

Supplementary Table 26

Supplementary Table 27

Supplementary materials

Dataset 1

## Data Availability

The datasets supporting the conclusions of this article are included within the article and its additional files. The RNAseq dataset was deposited onto GEO (accession number GSE287599). The proteomics and phosphoproteomics were deposited onto PRIDE (accession number PXD060126).
