# Supplementary material for: Multi-omics dissection of MAPK-driven senescence unveils therapeutic vulnerabilities in KIAA1549::BRAF-fusion pediatric low-grade glioma models
Source: Signal Transduct Target Ther. 2025 Jun 23;10:197. doi: 10.1038/s41392-025-02279-8 (PMC12185702; doi:10.1038/s41392-025-02279-8)

Supplementary Materials for

Multi-omics Dissection of MAPK-driven Senescence Unveils Therapeutic Vulnerabilities in KIAA1549::BRAF-fusion Pediatric Low-Grade Glioma Models

Romain Sigaud^1,2,3,4*^, Anja Stefanski^5^, Florian Selt^3,4,6,7^, Daniela Kocher^1,2,3,4,8^, Diren Usta^9,3,4,6^, Daniel Picard^10,11,12,13^, Isabel Büdenbender^3,4,6^, Marc Remke^10,11,12,13^, Stefan M. Pfister^3,6,7,14^, David T.W. Jones^3,6,15^, Tilman Brummer^16,17,18^, Olaf Witt^3,4,6,7^, Till Milde^1,2,3,4*^

Correspondence to: [Romain.Sigaud@med.uni-jena.de](mailto:Romain.Sigaud@med.uni-jena.de) and [Till.Milde@med.uni-jena.de](mailto:Till.Milde@med.uni-jena.de)

**This PDF file includes:**

Supplementary Materials and Methods

Supplementary Figures. S1 to S8

Captions for Supplementary Table 1 to 27

Captions for Dataset 1

Uncropped Western blot pictures

Supplementary Material and Methods

**LC-MS/MS phospho-/proteomics analysis of pLGG cell line**

*1. Sample preparation*

For protein extraction from frozen cell pellets, cells were lysed and homogenized in 3 times the amount of urea buffer (3 times the weight (mg) of the cells in μl) (30mM Tris Base 1M, 7M Urea, 2M Thiourea, adjusted to pH8.5) with a TissueLyser (Qiagen) and supernatants were collected after centrifugation for 15 min at 14,000 x g and 4°C. Protein concentration was determined by means of Pierce 660 nm Protein Assay (Thermo Fischer Scientific).

For LC-MS analysis a modified magnetic bead-based sample preparation protocol according to Hughes and colleagues was applied.2 Briefly, 300 µg total protein per sample were added to a mixture of magnetic beads (Sera-Mag SpeedBeads GE 45152105050250 and Sera-Mag SpeedBeads GE 65152105050250, mixed in equal parts) at a ratio of 1:10 protein / bead ratio and topped up with 200 μl H2O and 500 μl acetonitrile. The samples were incubated for 10 minutes at room temperature (RT) and 1000 rpm. Afterwards the supernatants were removed. The samples were washed twice with 1 ml of 70% ethanol and once with 1 ml of acetonitrile (ACN). To reduce the samples, 100 μl of 10 mM DTT (dithiothreitol) were added, and samples were incubated for 45 minutes at 56°C and 1000 rpm. After incubation, the supernatants were discarded. For alkylation, 100 μl of 55 mM IAA (iodoacetamide) was added, and the samples were incubated for 30 minutes at RT, in the dark, with 1000 rpm. Then, 250 μl of ACN (resulting in a final concentration of 70% ACN) was added, and the samples were incubated for 10 minutes at RT and 1000 rpm. After removing the supernatants, samples were washed twice with 1 ml of 70% ethanol and once with 1 ml of ACN. Next, 80 μl of freshly prepared 50 mM NH4HCO3 was added, and the samples were incubated for 10 minutes at RT and 1000 rpm. Finally, trypsin was added at a 1:100 ratio and the sample were digested overnight at 37°C and 1000 rpm. Extra-digestion was carried out by adding the same amount of trypsin and shaking at 37°C and 1000 rpm for another 4 h. Peptide elution was done by adding 100µL 2% DMSO. The supernatants were collected and 300 ng of each sample digest were subjected to LC-MS for whole proteome analysis.

*2. Phospho-enrichment*

A first enrichment of phosphopeptides was performed using an IMAC approach. For this, 5 µL of Ni-NTA (Qiagen) bead slurry per 100 µg of peptide were loaded onto ZipTip C18 columns and washed twice with 100 µL of 50% ACN in 1:20 NH3/H2O solution, followed by two additional washes with 200 µL of 80% ACN/0.1% TFA. The samples were then resuspended in 200 µL of 80% ACN/0.1% TFA and loaded onto the column, followed by a 30-minute incubation at room temperature. After incubation, the samples were centrifuged at 1000 rpm for 30 minutes (run-through). Next, the samples were washed three times with 200 µL of 80% ACN/0.1% FA and eluted twice with 50 µL of 50% ACN in 1:20 NH3/H2O solution. All centrifugation steps were performed at 6000 rpm for 2 minutes, except for the elution, which was centrifuged at 3000 rpm.

A second enrichment step was performed using TiO2. For this, 0.6 mg of TiO2 beads (5 µm, GL Science) per 100 µg of protein were used, conditioned according to the manufacturer’s protocol, and the run-through from the previous IMAC step was added. After adding the sample, the mixture was incubated for 30 minutes on an overhead shaker at room temperature. The samples were then transferred to prepared C8 tips and centrifuged at 6000 rpm for 1 minute. The flow-through was collected, and the samples were washed three times with the following steps:

1. Add 300 µL of binding buffer (1 M glycolic acid in 80% ACN, 5% TFA), centrifuge at 6000 rpm for 1 minute.

2. Add 200 µL of wash buffer (80% acetonitrile, 1% TFA), centrifuge at 6000 rpm for 1 minute.

3. Add 200 µL of wash buffer (80% acetonitrile, 1% TFA), centrifuge at 6000 rpm for 1 minute.

For elution, the samples were treated with 10 µL of 100% TFA and 50 µL of elution buffer (5% ammonium hydroxide, pH 12), and centrifuged at 3200 rpm for 5 minutes.

Subsequently, 300 µL of binding buffer was added and centrifuged at 4600 rpm for 8 minutes. The flow-through was then added, and the samples were centrifuged three times at 3000 rpm for 3 minutes. This washing and elution process was repeated twice.

*3. LC-MS/MS analysis*

For the LC-MS acquisition an Orbitrap Fusion Lumos Tribrid Mass Spectrometer (Thermo Fisher Scientific) coupled to an Ultimate 3000 Rapid Separation liquid chromatography system (Thermo Fisher Scientific, Idstein, Germany) equipped with an Acclaim PepMap 100 C18 column (75 µm inner diameter, 25 cm length, 2 µm particle size from Thermo Fisher Scientific) as separation column and an Acclaim PepMap 100 C18 column (75 µm inner diameter, 2 cm length, 3 µm particle size from Thermo Fisher Scientific) as trap column were used. A LC-gradient of 180 min was applied. Survey scans were carried out over a mass range from 200 - 2,000 m/z at a resolution of 120,000. The target value for the automatic gain control was 250,000 and the maximum fill time 60 ms. Within a cycle time of 2 s the most intense peptide ions (excluding singly charged ions) were selected for fragmentation. Peptide fragments were analyzed in the ion trap using a maximal fill time of 50 ms and automatic gain control target value of 10,000 operating in rapid mode. Already fragmented ions were excluded for fragmentation for 60 seconds.

*4. Data analysis*

Data analysis was performed with Proteome Discoverer (version 2.4.1.15, Thermo Fisher Scientific). All RAW files were searched against the human Swissprot database (Download: 23.01.2020) and the Maxquant Contaminant database (Download: 20.01.2021), applying a precursor mass tolerance of 10 ppm and a mass tolerance of 0.6 Da for fragment spectra. Methionine oxidation, N-terminal acetylation, N-terminal methionine loss and N-terminal methionine loss combined with acetylation as well as phosphorylation on S,T,R were considered as variable modification, carbamidomethylation as static modification. Tryptic cleavage specificity was set to a maximum of two missed cleavage sites. Labelfree quantification was performed using standard parameters within the predefined workflow. Post processing, proteins were filtered to 1% FDR and a minimum of 2 identified peptides per protein.


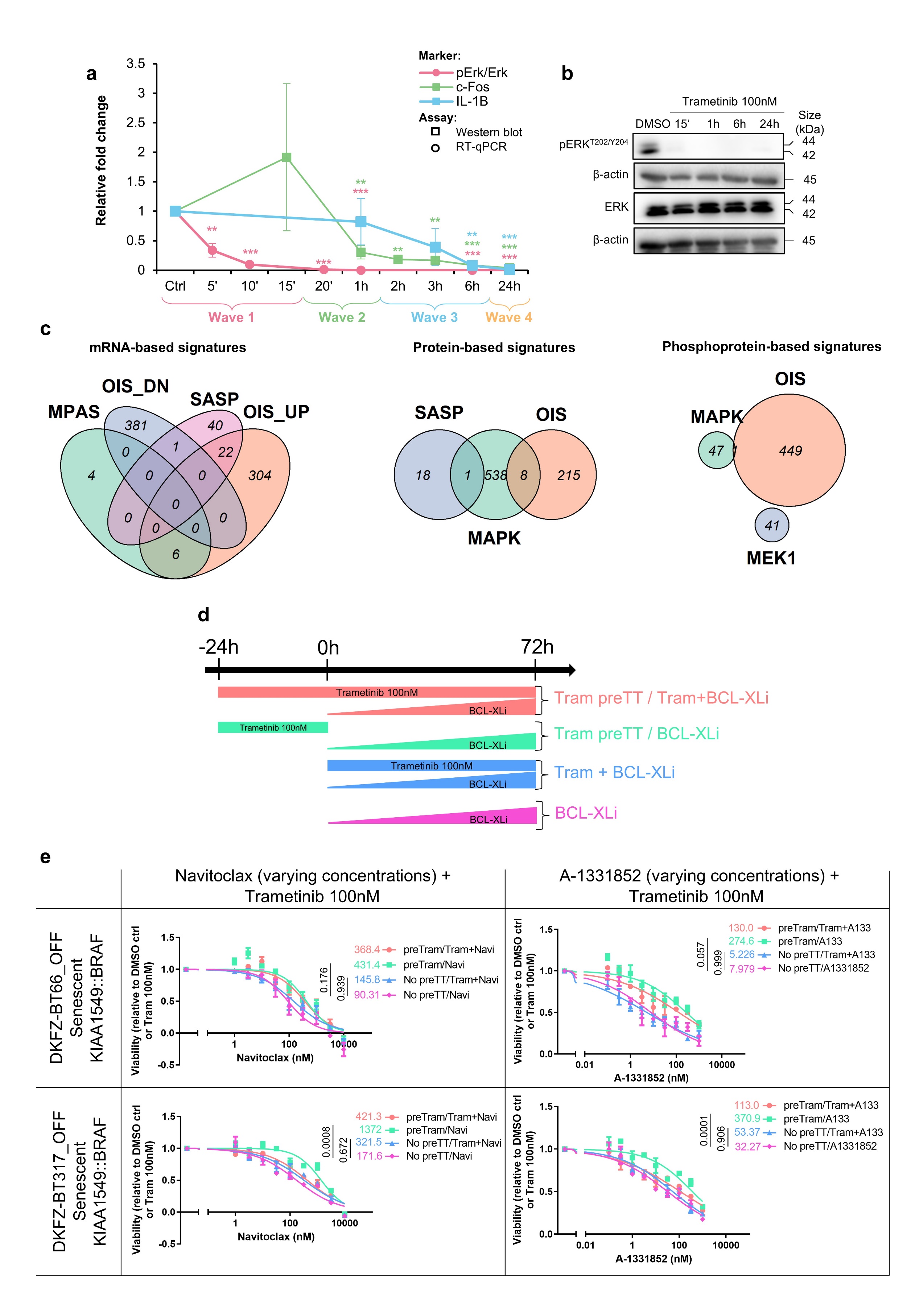


Figure. S1.

**Multi-omics samples’ QC, MAPK/OIS/SASP signatures overlap and dose-response curves**

**a.** Regulation of key molecules representative for each wave of activation (pERK – phosphoprotein by Western blot – wave 1; c-FOS – mRNA by RT-qPCR – wave 2; IL-1B – mRNA by RT-qPCR – wave 3) upon 100 nM trametinib treatment. Data are shown as mean +/- SD of 3 independent biological replicates. One-sample t-test, ** adj. p-val < 0.01, *** adj. p-val < 0.001, not significant if not specified. **b.** Western blot analysis of pERK Y202/T204 in the generated omics samples. QC to validate trametinib treatment efficacy before further sample processing. **c.** Venn diagrams depicting the overlap between the different signatures used to detect MAPK/OIS/SASP activity/enrichment. **d.** Schematic depiction of the treatment schedule applied on the senescent DKFZ-BT66 and DKFZ-BT317. **e.** Dose-response curves for the two senolytics navitoclax and A-1331852 in the senescent DKFZ-BT66 and DKFZ-BT317 in the corresponding treatment schedule. Data are shown as mean +/- SD. The data depict the results from n = 3 independent biological replicates. Significance was tested for the pairs indicated in the figure, adjusted p-values were calculated by one-way ANOVA followed by Sidak’s multiple comparisons test.


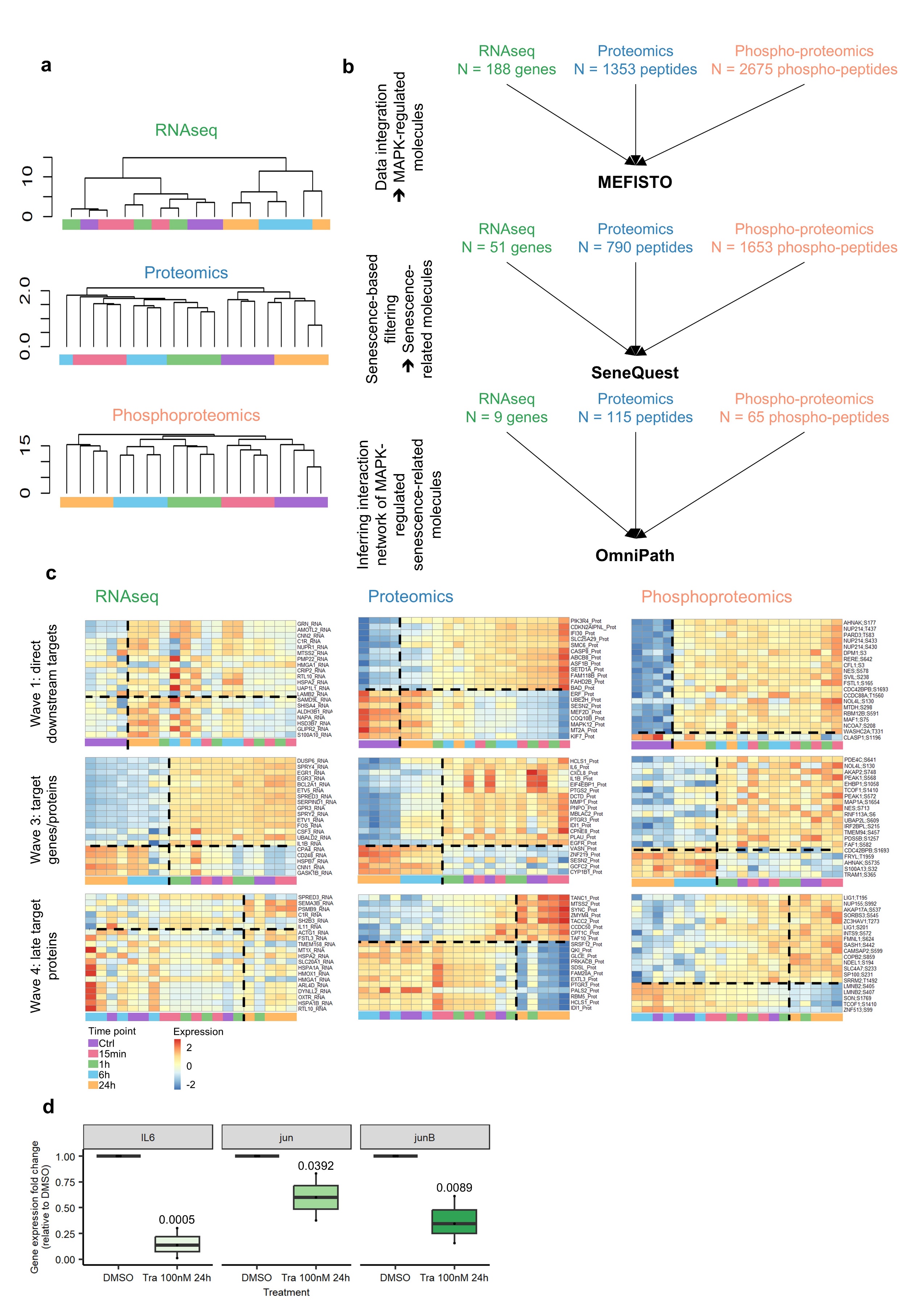


Figure. S2.

**Detailed results from the MEFISTO analysis**

**a.** Unsupervised hierarchical clustering illustrating samples clustering upon MAPKi through time, before data integration in MEFISTO. **b.** Schematic depiction of the data processing pipeline. MEFISTO was used to identify key molecules regulated by the MAPK pathway, filtered using the SeneQuest database to only retain the molecules involved in senescence, and mapped as a pathway using the OmniPath database. **c.** Heatmaps depicting the top 20 most up-/downregulated molecules from each factors related to wave 1, 3 and 4. **d.** Boxplot depicting the relative fold change expression by RT-qPCR for *IL6*, *jun*, *junB* in DKFZ-BT66 samples upon treatment with DMSO or trametinib 100nM for 24h. p-values (one-way ANOVA followed by the Tukey's ‘Honest Significant Difference’) are depicted. The experiment was conducted with n=3 independent biological replicates.


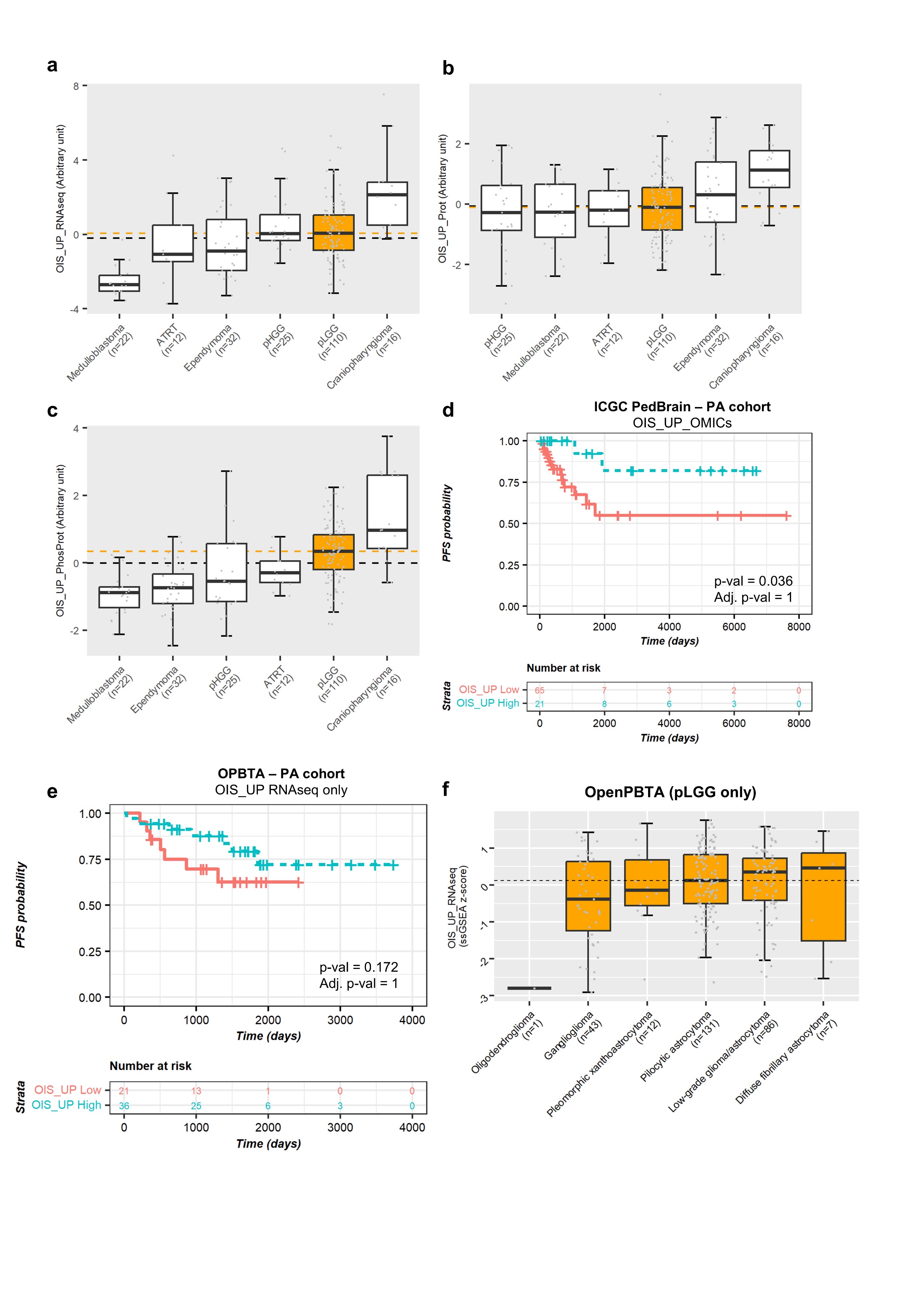


Figure. S3.

**Validation of the MAPK/OIS/SASP molecules in primary pLGG samples**

**a-c.** Boxplot depicting the enrichment of the OIS_UP genes (**a**), proteins (**b**) and phosphoproteins (**c**) (z-score sum, arbitrary unit) in n = 6 pediatric glioma entities from the ProTrack Pediatric Brain Tumor dataset from Petralia et al. Black dashed line depicts overall median, orange dashed line depicts pLGG median. **d.** Kaplan-Meier curve of primary PA samples from the ICGC PedBrain cohort grouped based on their enrichment for the OIS_UP molecules from all omics layers (p-value from log-rank test, and adjusted p-value corrected by Bonferroni method after multiple testing to identify the optimal cut-off with the best raw p-value). **e.** Kaplan-Meier curve of primary BRAF-driven PA samples from the OPBTA cohort grouped based on their enrichment for the OIS_UP molecules from all omics layers (p-value from log-rank test, and adjusted p-value corrected by Bonferroni method after multiple testing to identify the optimal cut-off with the best raw p-value). **f.** Boxplot depicting the ssGSEA z-score of the OIS_UP genes from the RNAseq layer in n = 285 pediatric low-grade glioma samples split based on histopathological diagnosis, as indicated in the Open Pediatric Brain Tumor Atlas.


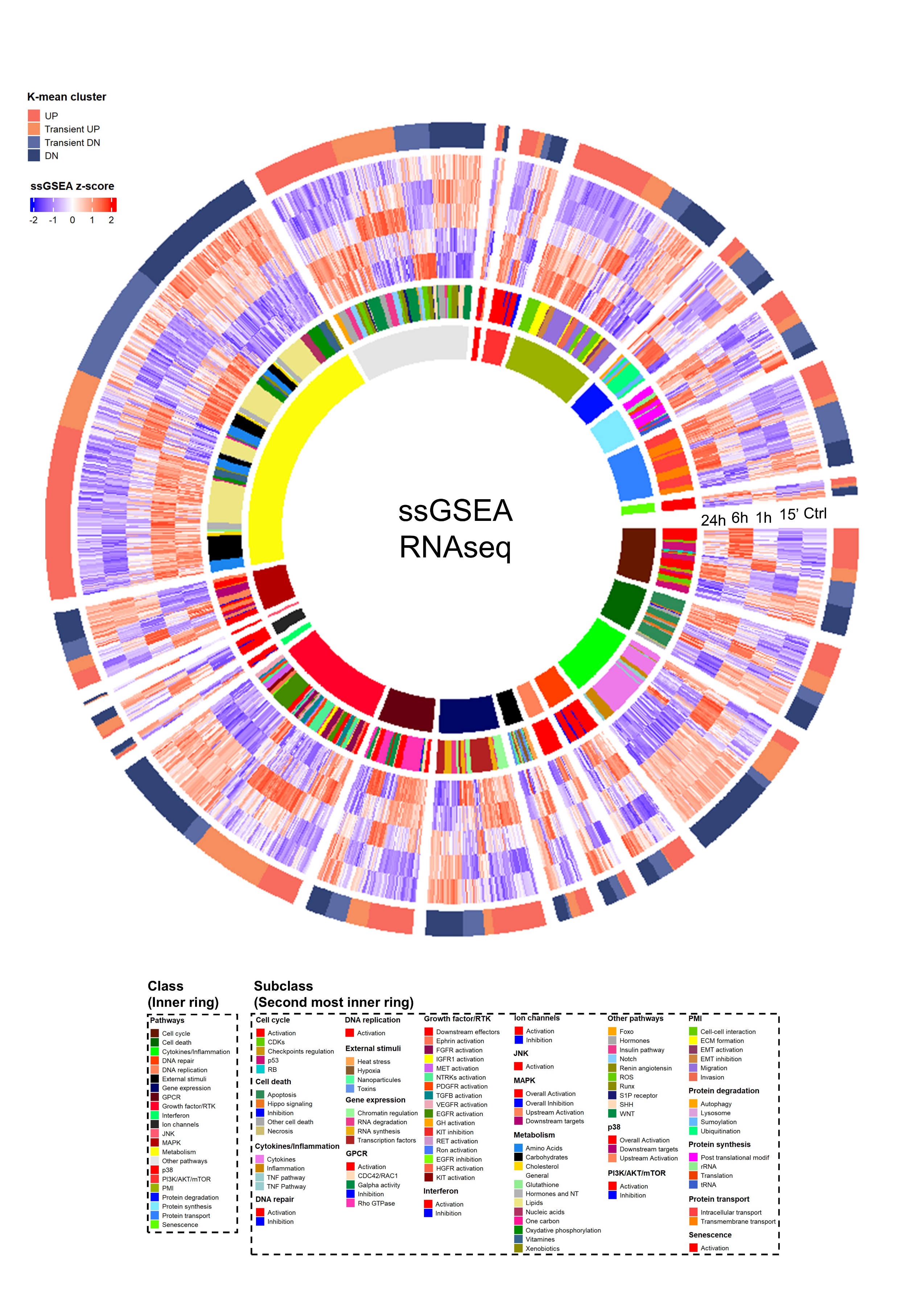


Figure. S4.

**Pathways differentially regulated upon MAPKi treatment on the mRNA level.** Circular heatmap depicting the gene signatures differentially enriched through time upon MAPKi treatment (trametinib 100 nM) in the senescent DKFZ-BT66 cells. K-means clusters were identified in the longitudinal k-mean clustering in Suppl. Fig. S7. A list of signatures specific for each type of omics layer was used to ensure accuracy.


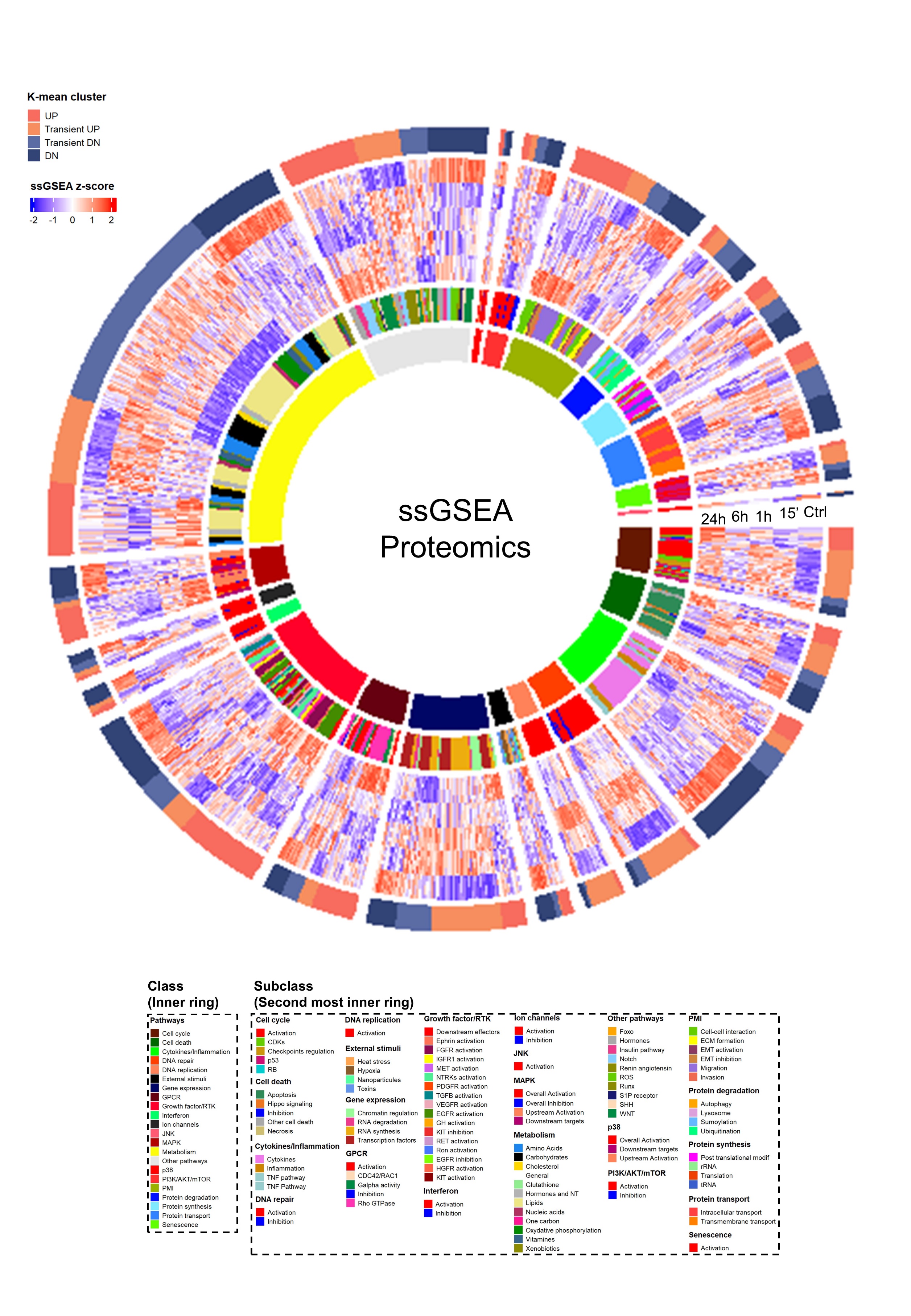


Figure. S5.

**Pathways differentially regulated upon MAPKi treatment on the protein level.**Circular heatmap depicting the protein signatures differentially enriched through time upon MAPKi treatment (trametinib 100 nM) in the senescent DKFZ-BT66 cells. K-means clusters were identified in the longitudinal k-mean clustering in Suppl. Fig. S7. A list of signatures specific for each type of omics layer was used to ensure accuracy.


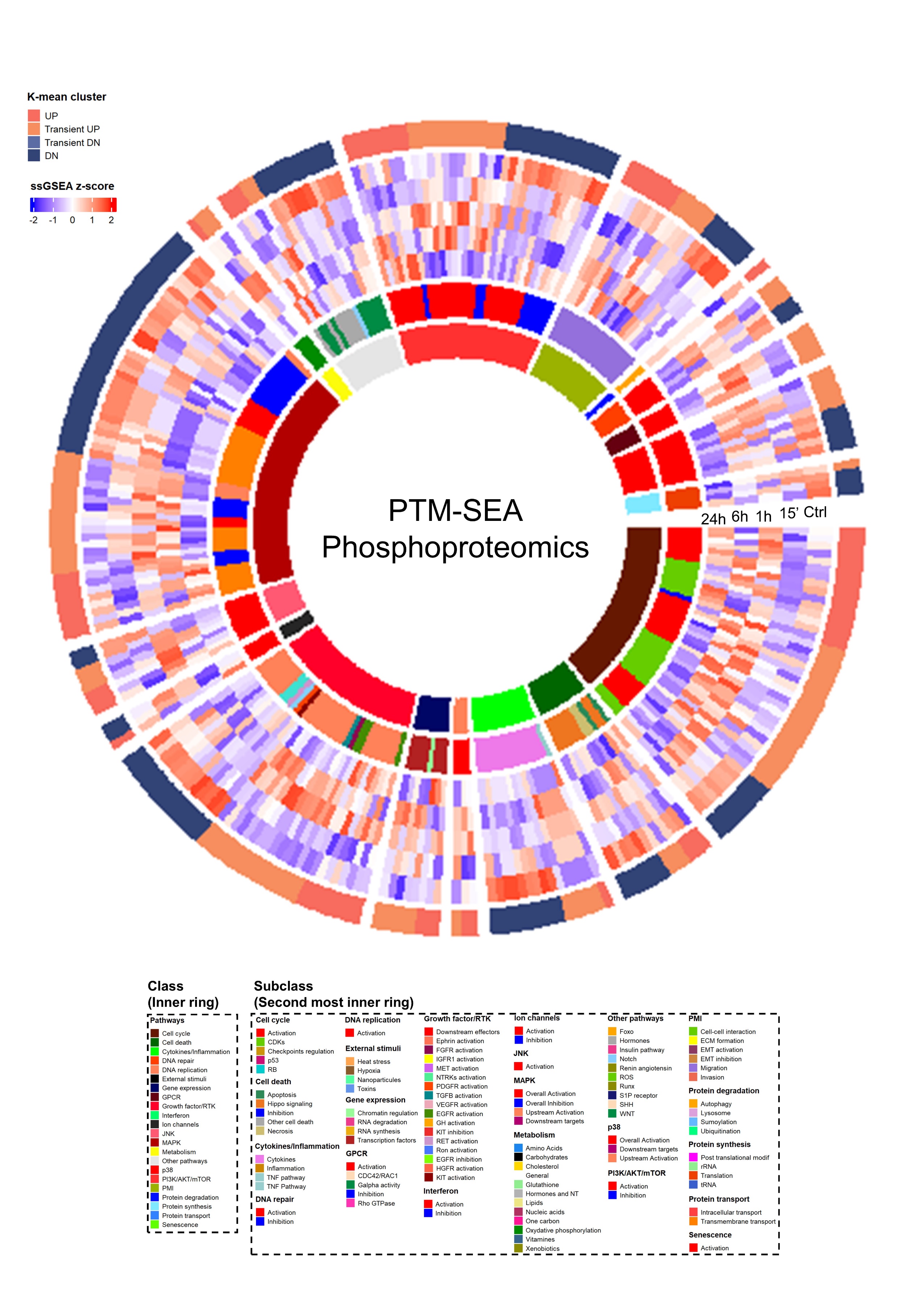


Figure. S6.

**Pathways differentially regulated upon MAPKi treatment on the phosphoprotein level.**Circular heatmap depicting the phosphoprotein signatures differentially enriched through time upon MAPKi treatment (trametinib 100 nM) in the senescent DKFZ-BT66 cells. K-means clusters were identified in the longitudinal k-mean clustering in Suppl. Fig. S7. A list of signatures specific for each type of omics layer was used to ensure accuracy.


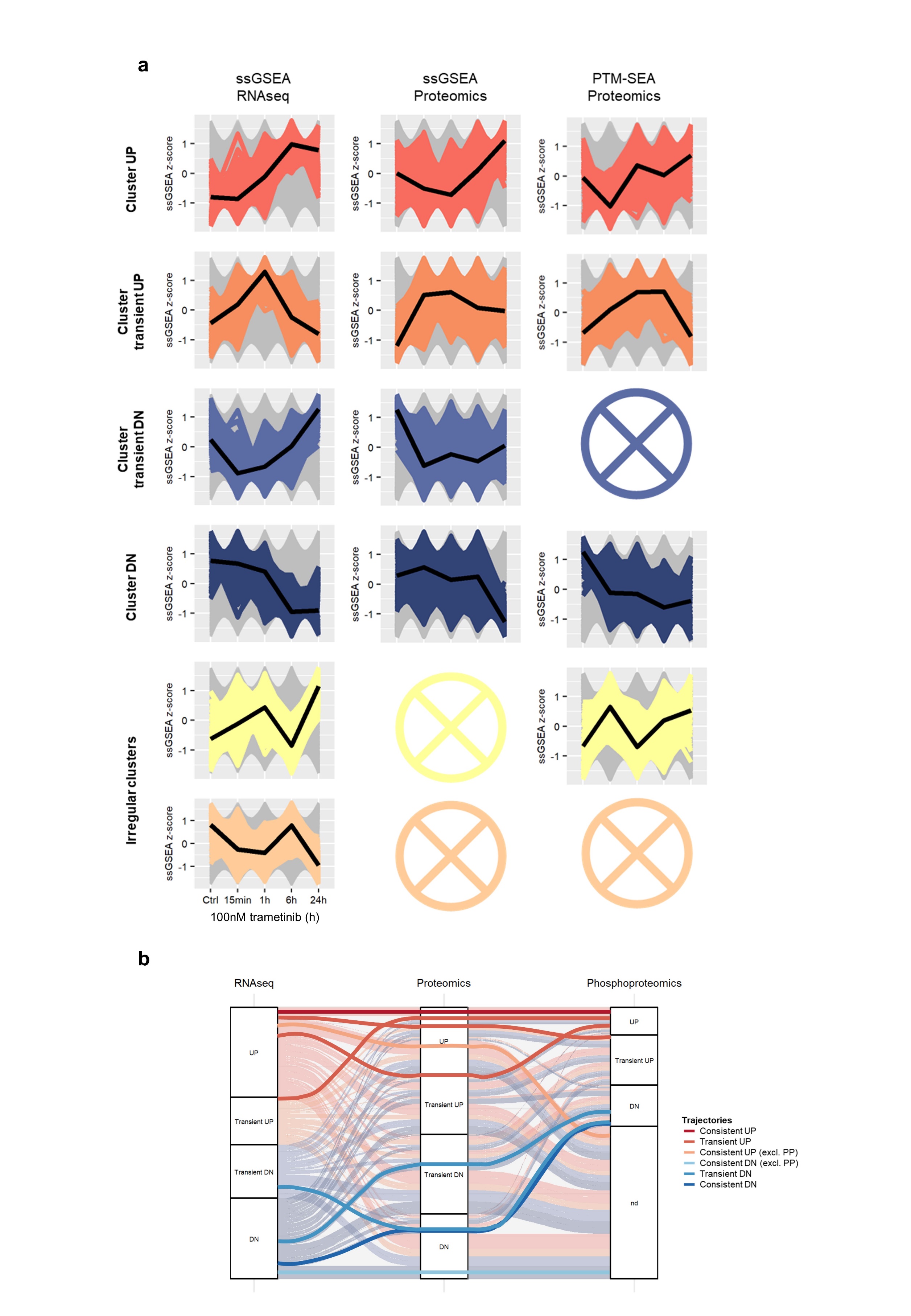


Figure. S7.

**Identification of consistently regulated pathway across omics layers upon MAPKi**

**a.** Longitudinal k-means clustering of differentially regulated signatures from the RNAseq, proteomics and phosphoproteomics datasets upon 100 nM trametinib treatment. Highlighted in color are all the pathways whose expression follow the main regulation pattern (black line) identified by longitudinal k-means clustering. **b.** Alluvial plot depicting the regulation of every given signature across all omics layers. To ensure robustness, only the signatures consistently regulated in at least n = 2 omics layers (bold curves) were kept for further analysis.


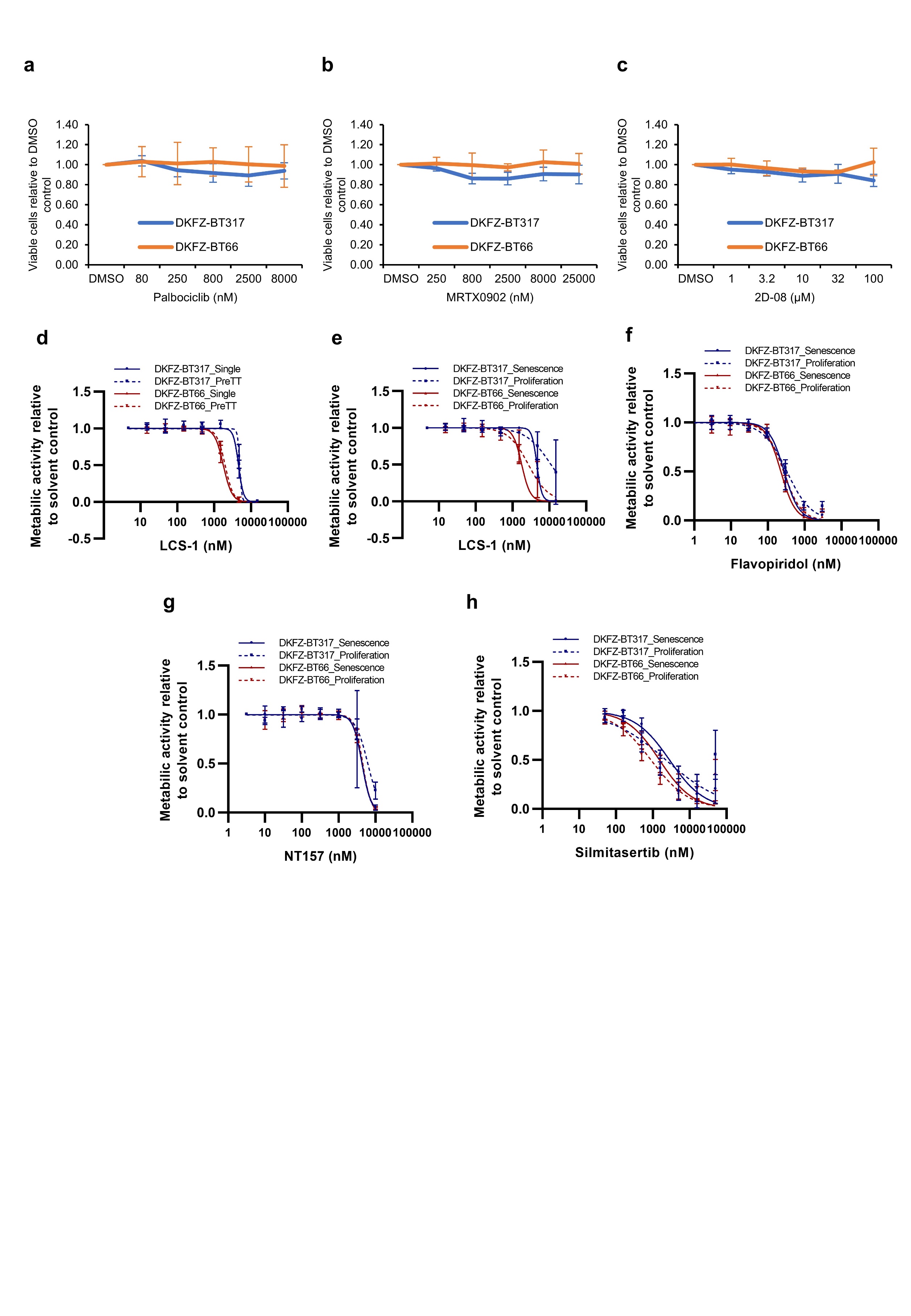


Figure. S8.

**Validation of key drugs via cell counts or dose-response curves in pLGG cell lines.**

**a-c.** Graphs depicting cell counts of senescent DKFZ-BT66 and DKFZ-BT317 upon 72 h treatment with the hypothesized growth promoting drugs palbociclib (**a**), MRTX0902 (**b**) or 2-D08 (**c**). **d.** Dose response curve for the validation of LCS-1 as a MAPK-dependent senolytic drug. **e-h.** Dose response curve for the validation of the hypothesized MAPK-(in)dependent cytotoxic drugs in the senescence vs proliferating DKFZ-BT66 and DKFZ-BT317. The graphs depict the results from n = 3 independent biological replicates, as mean +/- SD.

Supplementary Table 1. (separate file)

Raw quantification of WB/qPCR of key MAPK wave markers

Supplementary Table 2. (separate file)

Differentially regulated genes, proteins and phosphopeptides in the omics dataset

Supplementary Table 3. (separate file)

Gene-/protein-/phosphoprotein-based signatures related to MAPK activity, OIS and SASP.

Supplementary Table 4. (separate file)

ssGSEA score for MAPK/OIS/SASP gene, protein and phosphoprotein signatures in the senescent DKFZ-BT66 cells

Supplementary Table 5. (separate file)

Metabolic activity in the pLGG cell lines upon MEKi+senolytic treatment, and upon treatment with the newly identified drugs

Supplementary Table 6. (separate file)

Genes, proteins and phosphopeptides that passed pre-selection threshold and used in the MEFISTO model

Supplementary Table 7. (separate file)

List of effectors identified in the MEFISTO model and their regulation upon MAPKi in our omics dataset

Supplementary Table 8. (separate file)

MAPK/OIS signatures for each omics layers

Supplementary Table 9. (separate file)

List of senescence-related genes from the SeneQuest database, and 100 random lists of UP and DN regulated genes from the SeneQuest list

Supplementary Table 10. (separate file)

Output from the STRING analysis of 100 randomly selected gene lists from the Senequest database

Supplementary Table 11. (separate file)

ssGSEA scores for the OIS signatures in a panel of 9 pLGG cell lines

Supplementary Table 12. (separate file)

ssGSEA scores for the OIS signatures in primary pLGG samples from the ProTrack Pediatric Brain Tumor dataset

Supplementary Table 13. (separate file)

ssGSEA scores for the OIS signatures in primary pLGG samples from the Open Pediatric Brain Tumor Atlas

Supplementary Table 14. (separate file)

ssGSEA scores for the OIS signatures in primary pLGG samples from the ICGC PedBrain cohort

Supplementary Table 15. (separate file)

ssGSEA scores of gene, protein and phosphoprotein specific signatures in our omics dataset

Supplementary Table 16. (separate file)

List of signatures and their regulation pattern through time and omics layers

Supplementary Table 17. (separate file)

List of signatures consistently regulation upon MAPKi in our omics dataset

Supplementary Table 18. (separate file)

GSEA results from the RNAseq layer, comparing 24h trametinib 100nM vs control, with the HALLMARK signature set

Supplementary Table 19. (separate file)

List of 35 drugs tested in our mini-drug screen

Supplementary Table 20. (separate file)

Metabolic activity measured from the mini-drug screen

Supplementary Table 21. (separate file)

List of seeding densities used throughout the study

Supplementary Table 22. (separate file)

Drugs used in the experiments with trametinib + BCL-XLi

Supplementary Table 23. (separate file)

List of antibodies and primers used in the study

Supplementary Table 24. (separate file)

Raw proteomics data used for PhosR correction

Supplementary Table 25. (separate file)

Raw phosphoproteomics data used for PhosR correction

Supplementary Table 26. (separate file)

Summary list of variance explained per factor for 15 MEFISTO iterations testing n=1 to n=15 factors to build the model

Supplementary Table 27. (separate file)

List of ssGSEA/PTM-SEA signatures used in the study and manually annotated for specific class and subclass

Data S1. (separate file)

Raw gene expression, protein and phosphopeptide abundance in our omics dataset

Uncropped Western blot pictures


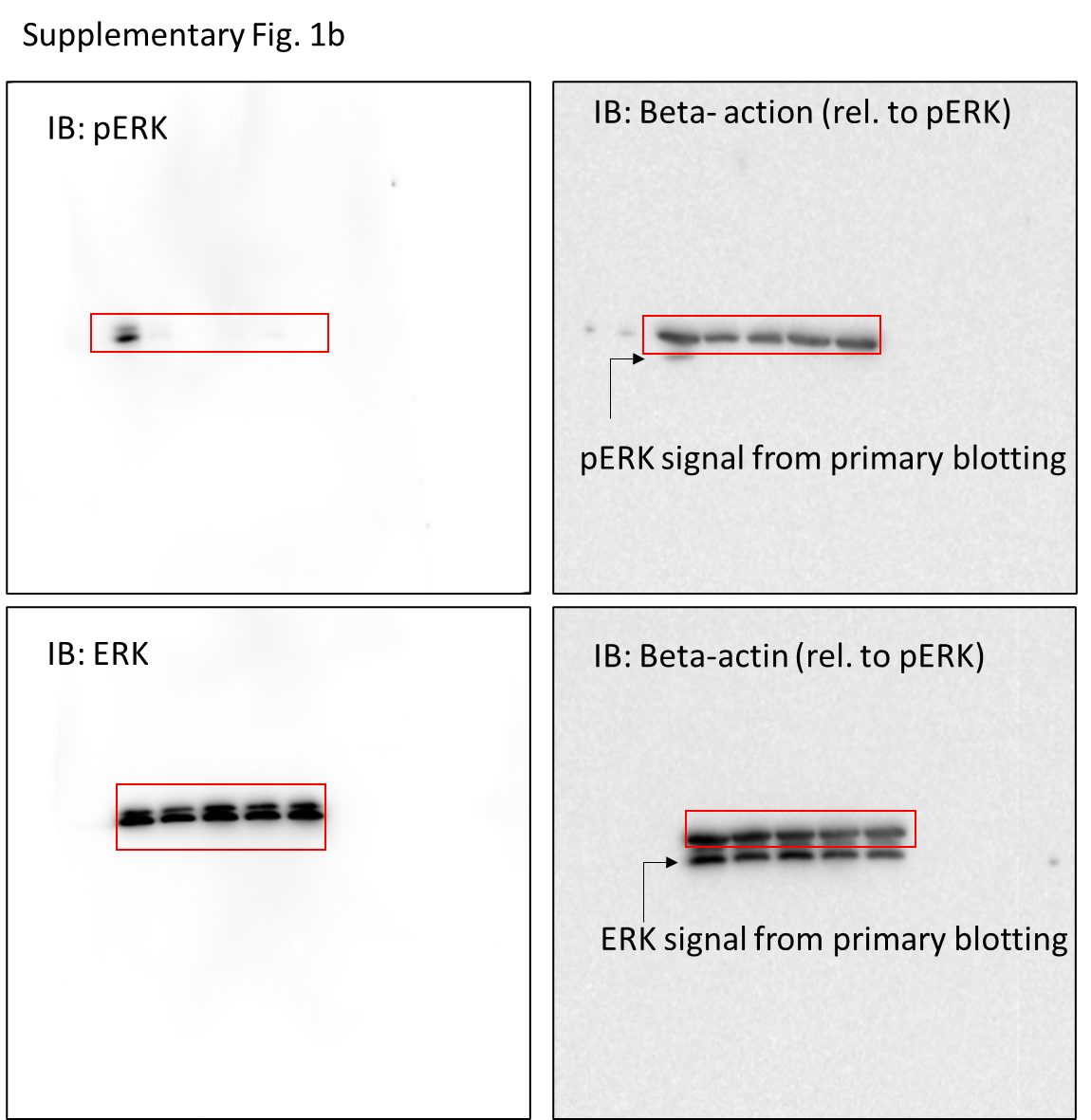

Supplement: Supplementary file 28 — Supplementary materials [file 41392_2025_2279_MOESM28_ESM.docx]
